# Highly efficient visible-light induced N-doped ZnO@g-C_3_N_4_ and S-doped ZnO@g-C_3_N_4_ photocatalysts for environmental remediation†

**DOI:** 10.1039/d3ra06488c

**Published:** 2024-01-03

**Authors:** Sukanya Borthakur, Riya Das, Purashri Basyach, Karanika Sonowal, Lakshi Saikia

**Affiliations:** a Advanced Materials Group, Materials Sciences and Technology Division, CSIR-North East Institute of Science and Technology Jorhat 785006 Assam India l.saikia@gmail.com lakshi_saikia@yahoo.com lsaikia@neist.res.in +91 0376 2370011 +91 9957031635; b Academy of Scientific and Innovative Research Ghaziabad UP 201002 India

## Abstract

Facile, cost-effective and eco-friendly synthesis of N-doped ZnO@g-C_3_N_4_ and S-doped ZnO@g-C_3_N_4_ photocatalysts towards efficient degradation of environmental pollutants was achieved. The as-synthesized 2 wt% N-doped ZnO@g-C_3_N_4_ and 2 wt% S-doped ZnO@g-C_3_N_4_ achieved 96.2% and 90.4% degradation efficiencies towards crystal violet (100 ppm) within 45 min irradiation and 99.3% and 92.3% photocatalytic degradation efficiencies towards brilliant green (100 ppm) dye within 30 min irradiation, respectively, under a normal 90 W LED light instead of an expensive commercial light source. Moreover, the N-doped ZnO@g-C_3_N_4_ and S-doped ZnO@g-C_3_N_4_ nanocomposites showed excellent stability in the photodegradation of crystal violet and brilliant green dyes. The modification made on ZnO by doping with nitrogen and sulphur enhances the visible-light absorption as well as the separation of photoexcited charge carriers. The active radicals ˙OH and ˙O_2_^−^ are both identified to play important roles in the photodegradation of crystal violet and brilliant green.

## Abstract 2

Facile, cost-effective and eco-friendly synthesis of N-doped ZnO@g-C_3_N_4_ and S-doped ZnO@g-C_3_N_4_ photocatalysts towards efficient degradation of environmental pollutants was achieved.

## Graphical abstract

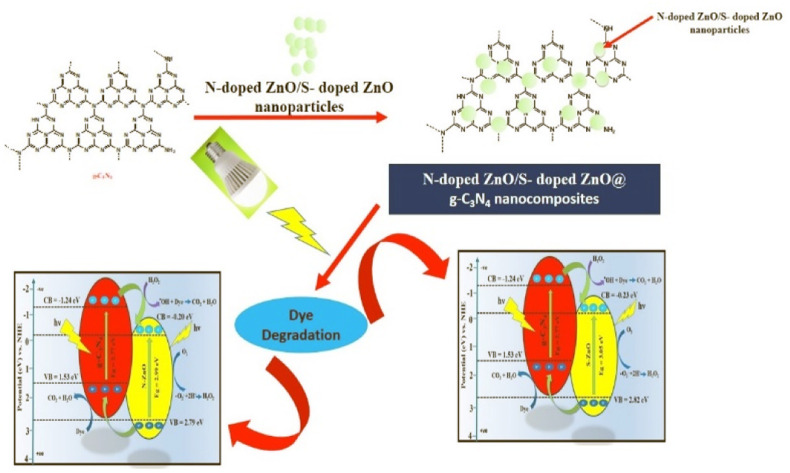

Fast industrialization, urbanization, population growth and the ongoing development of technologies have created severe concerns about increasing environmental pollution on a global scale. Among the different pollutants, organic molecules like dyes, phenolic compounds, heavy metals, fertilizers, herbicides, pesticides, pharmaceutical pollutants, pathogens, industrial effluents, sewage, and particulate matter have been identified as the main concerning water pollutants to date.^1,2^ Organic synthetic dyes used in various manufacturing industries, such as the textile, paper, cosmetic, food and consumer electronic manufacturing sectors, with an annual global production of about 0.7 million tons, constitute a considerable proportion of the environmental pollution because their non-biodegradability and high toxicity lead to the destruction of the environment.^1^ Synthetic dye contamination in the aqueous stream from different industrial sources is a matter of great concern because of its devastating effects on human health and the ecosystem. Worldwide, 0.28 million tons of non-biodegradable textile dye residues are discharged annually into the aquatic environment.^2^ The direct discharge of these organic contaminants from various industrial sources into the aquatic environment may lead to acute and chronic health issues in humans, animals and aquatic systems. Therefore, the design and development of green technology for the purification of contaminated surface and ground water sources has become vitally important for a safer environment. In general, these dyes have been found to be resistant to biological and physical treatment technologies. In this regard, the advanced oxidation process (AOP) involving the photo-catalytic degradation of dyes using semiconductor nanoparticles is versatile and has higher efficiency compared to other conventional oxidation processes for the removal of dye pollutants from aqueous streams.

Recently, semiconductor photocatalysis has attracted remarkable attention due to its clean and economic process for the complete degradation of organic contaminants. Among the semiconductors, ZnO is a promising material for advanced applications because of its unique features, including environmental stability, wide band gap, low cost, non-toxicity and unique chemical and physical properties.^3,4^ However, the wide band gap and low charge separation characteristic limit the photocatalytic activity of ZnO.^5^ To extend its response into visible light and improve its electron–hole separation efficiency, many attempts have been made, such as doping with metal and non-metal ions, controlling the morphology, forming a heterojunction with other materials.^6,7^ Among these, metal and non-metal doping have drawn much interest.^8^ Of the non-metal elements, N has proven to be a suitable p-dopant because the ionic radius and electronegativity of N are very close to those of O; hence, N doping may result in minimum strain in ZnO.^9^ Conversely, doping S is an efficient method for enhancing the visible-light activity of ZnO. Because of its large electronegativity as well as the size difference between S (*r*^2−^ = 0.18 nm) and O (*r*^2−^ = 0.14 nm), S-doping in ZnO helps to modulate the electrical and optical properties of ZnO.^10^ Therefore, both N- and S-doping can be considered potential pathways for extending the visible-light absorption of ZnO.

Graphitic carbon nitride (g-C_3_N_4_), a great potential material, has received significant interest because of its probable applications in environmental pollutant degradation, catalysing water splitting for H_2_ evolution, transesterification, hydrogen production, oxygen reduction and reducing carbon dioxide under visible-light irradiation due to its moderate band gap (2.7 eV), cost-effectiveness, unique electronic and optical properties, high stability and earth-abundant nature.^11,12^ However, the photocatalytic performance of this semiconductor photocatalyst is still limited because of its small specific surface area and fast recombination of charge carriers.^12^ This situation can be ameliorated through appropriate modification protocols, such as doping with organic or inorganic elements, coupling with other semiconductor components, and building heterostructures with another semiconductor.^13^ Constructing a suitable heterojunction composite by semiconductor coupling with a suitable band structure is an efficient way to improve the photo-excited charge separation.^14^ To date, various semiconductors have been investigated to construct g-C_3_N_4_ based composite photocatalysts such as ZnFe_2_O_4_/g-C_3_N_4_,^15^ WO_3_/g-C_3_N_4_,^12^ SnO_2_/g-C_3_N_4_,^16^ Fe_2_O_3_/g-C_3_N_4_,^17^ Ag_3_PO_4_/g-C_3_N_4_,^18^ ZnWO_4_/g-C_3_N_4_,^19^ SrTiO_3_/g-C_3_N_4_,^20^ CuCr_2_O_4_/g-C_3_N_4_,^21^ TiO_2_/g-C_3_N_4_,^22^ ZnO/g-C_3_N_4_,^23^ N–TiO_2_/g-C_3_N_4_,^24^ S–TiO_2_/g-C_3_N_4_,^25^*etc.*, to improve the visible-light-driven photocatalytic efficiency. Suneel *et al.* observed that N–ZnO/g-C_3_N_4_ nanocomposite was an efficient visible-light active photocatalytic material towards the degradation of toxic pollutants like rhodamine B, thiadiazol, nitrobenzene and tetracycline.^13^ Santosh *et al.* reported that core–shell nanoplates of N-doped ZnO/g-C_3_N_4_ showed improved photocatalytic activity towards the visible-light assisted degradation of rhodamine B.^9^ Xi-Yu *et al.* prepared a g-C_3_N_4_/S-doped ZnO nanocomposite *via* a one-pot method using sodium lignosulfonate which showed efficient photodegradation towards phenol and methylene blue.^26^

This article describes a facile eco-friendly synthesis strategy for N-doped ZnO@g-C_3_N_4_ and S-doped ZnO@g-C_3_N_4_ nanocomposites with remarkably improved visible-light induced catalytic efficiency. The photocatalysts were prepared by a simple cost-effective sonication-assisted route with different loadings of N-doped ZnO and S-doped ZnO nanoparticles. The excellent performance of N-doped ZnO@g-C_3_N_4_ and S-doped ZnO@g-C_3_N_4_ nanocomposites was assessed for the photodegradation of crystal violet and brilliant green dyes under 90 W LED light irradiation. The use of a 90 W LED light having a luminous intensity of 8060 candela instead of any commercially available expensive, high intensity light source for photocatalytic degradation represents an energetically economical alternative. The extended adsorption in the visible-light region and the higher separation efficiency of photo-excited charge carriers promoted the photocatalytic activity under visible-light irradiation.

## Results and discussion

Zeta potential analysis was carried out to investigate the proper conditions for appropriate bonding between g-C_3_N_4_, N-doped ZnO nanoparticles and S-doped ZnO nanoparticles. The zeta potential value of g-C_3_N_4_ is −26.5 mV at neutral pH. On the other hand, N-doped ZnO and S-doped ZnO nanoparticles have positive zeta potential values of 25.9 mV and 27.3 mV, respectively, under acidic condition. This clearly confirms the spontaneous self-assembly between the negatively charged surface of g-C_3_N_4_ and the positively charged surfaces of N-doped ZnO and S-doped ZnO under acidic condition in the case of N-doped ZnO@g-C_3_N_4_ and S-doped ZnO@g-C_3_N_4_ nanocomposites.

**Fig. 1:**
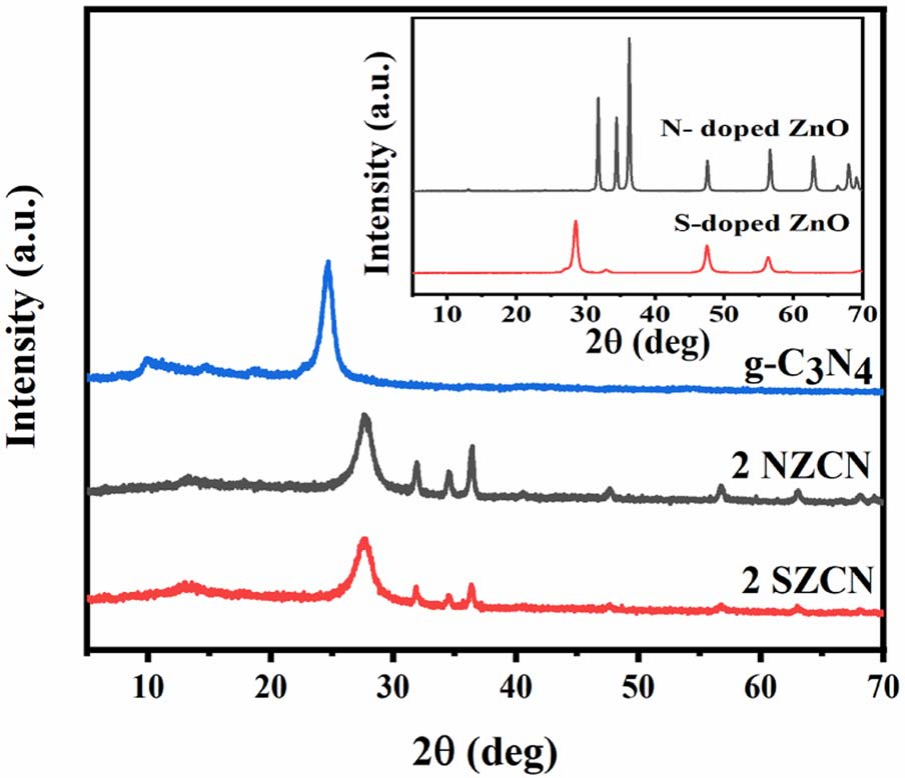
XRD patterns of 2 NZCN and 2 SZCN nanocomposites and g-C_3_N_4_. Inset: XRD patterns of N-doped ZnO and S-doped ZnO nanoparticles.

The XRD patterns of g-C_3_N_4_ and 2 NZCN and 2 SZCN nanocomposites are depicted in Fig. 1. The strong peak at 2*θ* = 27.18 corresponds to the (002) crystal phase, which is a characteristic interplanar stacking structure of graphitic material (JCPDS-87-1526, inset Fig. 1).^15^ In the case of N-doped ZnO and S-doped ZnO nanoparticles, the characteristic peaks correspond to the hexagonal wurtzite phase of ZnO without any impurity, suggesting that the crystal structures remain the same after the incorporation of nitrogen and sulphur, respectively (JCPDS-36-1451).^26,27^ The diffraction patterns of N-doped ZnO and S-doped ZnO are significantly broader than that of bare ZnO due to their decreasing crystallinity because of the introduction of N and S into the ZnO lattice. The XRD patterns show no extra diffraction peaks, indicating N-doped ZnO@g-C_3_N_4_ and S-doped ZnO@g-C_3_N_4_ to be two-phase nanocomposite hybrids.

The FT-IR spectra of pure g-C_3_N_4_, pure ZnO, N-doped ZnO, S-doped ZnO, and 2 NZCN and 2 SZCN nanocomposites are depicted in Fig. S1.† In the cases of ZnO, N-doped ZnO and S-doped ZnO, the peaks at 400–550 cm^−1^ correspond to the Zn–O stretching vibration.^26,27^ As shown in Fig. S1,† the IR absorption bands showed the typical molecular structure of pure g-C_3_N_4_. A broad peak centered at 3152 cm^−1^ corresponds to the N–H stretching vibration and the peak at 808 cm^−1^ is assigned to the stretching vibrational mode of the s-triazine ring of g-C_3_N_4_.^15^ The peaks ascribed to the stretching vibrations of the CN heterocycles are within the range of 1200–1650 cm^−1^ for the pure g-C_3_N_4_ nanosheets. It is observed that all the major characteristic peaks of g-C_3_N_4_, N-doped ZnO and S-doped ZnO are retained in the FT-IR spectra of 2 NZCN and 2 SZCN nanocomposites. From the figure, it was seen that, in the cases of 2 NZCN and 2 SZCN, the major distinctive bands of g-C_3_N_4_ are retained in the nanocomposites, which indicates that the structural features of g-C_3_N_4_ are retained after the hybridization process. Furthermore, the main characteristic peaks of g-C_3_N_4_ in the 2 NZCN and 2 SZCN nanocomposites slightly shift to higher wave numbers, which is attributable to the extended conjugated system in the nanocomposites.^27^

TGA was carried out from room temperature to 800 °C at a heating rate of 10 °C min^−1^ under N_2_ flow in order to calculate the contents of N-doped TiO_2_ and S-doped TiO_2_ nanoparticles on the g-C_3_N_4_ nanosheets in the as-synthesized N-doped ZnO@g-C_3_N_4_ and S-doped ZnO@g-C_3_N_4_ nanocomposites. The TG/DTA plots of as-prepared pure g-C_3_N_4_ and N-doped ZnO@g-C_3_N_4_ and S-doped ZnO@g-C_3_N_4_ nanocomposites are depicted in Fig. S2.† For bare g-C_3_N_4_, a weight loss occurred from 500 °C to 700 °C due to the decomposition or condensation of g-C_3_N_4_.^28^ For the N-doped ZnO@g-C_3_N_4_ and S-doped ZnO@g-C_3_N_4_ nanocomposites, the stability of the nanocomposites significantly decreased due to the oxidation and decomposition of g-C_3_N_4_ in the composite materials.^30^ The thermal stability decreased from 482 °C to 694 °C and from 485 °C to 698 °C for the N-doped ZnO@g-C_3_N_4_ and S-doped ZnO@g-C_3_N_4_ nanocomposites, respectively, compared to g-C_3_N_4_, indicating the close contact between N-doped ZnO and S-doped ZnO with the g-C_3_N_4_ nanosheets. The residual weight fractions of the 2 NZCN and 2 SZCN nanocomposites were found to be 1.3% and 2.6%, respectively, and these are considered to be the contents of N-doped ZnO and S-doped ZnO in the 2 NZCN and 2 SZCN nanocomposites, respectively.

**Fig. 2:**
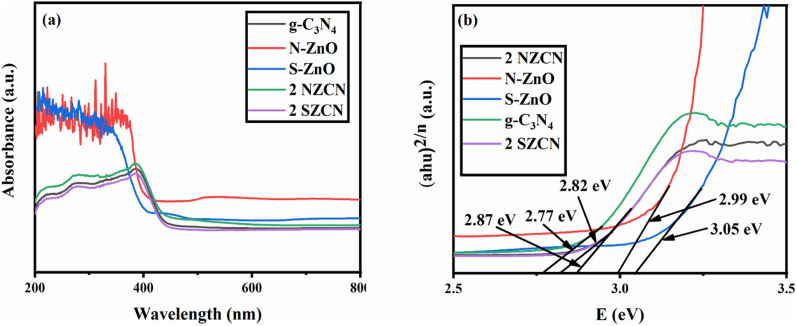
(a) UV-vis spectra and (b) band gap energies of g-C_3_N_4_, N-doped ZnO, S-doped ZnO, and 2 NZCN and 2 SZCN nanocomposites.

UV-vis diffuse reflectance spectroscopy (DRS) was carried out to investigate the visible-light absorption of the N-doped ZnO@ g-C_3_N_4_ and S-doped ZnO@g-C_3_N_4_ nanocomposite photocatalysts. The absorption edge of pure g-C_3_N_4_ is around 452 nm (Fig. 2(a)). The absorption of N-doped ZnO@g-C_3_N_4_ and S-doped ZnO@g-C_3_N_4_ photocatalysts in the visible-light region is considerably enhanced and a red shift compared to bare g-C_3_N_4_ appeared due to the interaction of the g-C_3_N_4_ nanosheets with the N-doped ZnO and S-doped ZnO NPs in the nanocomposites.^26^ The wider light absorption region of the as-prepared nanocomposites is able to effectively produce more photo-generated charge carriers for better photocatalytic activity under visible-light absorption.^30^

The band gap energy of samples was evaluated according to eqn (1),

1 *αhυ* = *A* (*hυ* − *E*_g_)^*n*/2^

where *α*, *h*, *υ*, *E*_g_ and *A* denote the absorption coefficient, Planck's constant, light frequency, band gap energy and a constant, respectively. The value of *n* can be obtained from the type of optical transition in the semiconductor (*n* = 1 for direct transition and *n* = 4 for indirect transition).^15^ In case of g-C_3_N_4_, and ZnO, the values of *n* are found to be 1 and 4 respectively. From the plot of (*αhυ*)^2^*versus* energy (*hυ*) (Fig. 2(b)), the *E*_g_ of pure g-C_3_N_4_ and N-doped ZnO@g-C_3_N_4_ and S-doped ZnO@g-C_3_N_4_ nanocomposites are found to be 2.75 eV, 2.72 eV and 2.81 eV, respectively, which are in favour of a visible-light response of the photocatalysts.

**Fig. 3:**
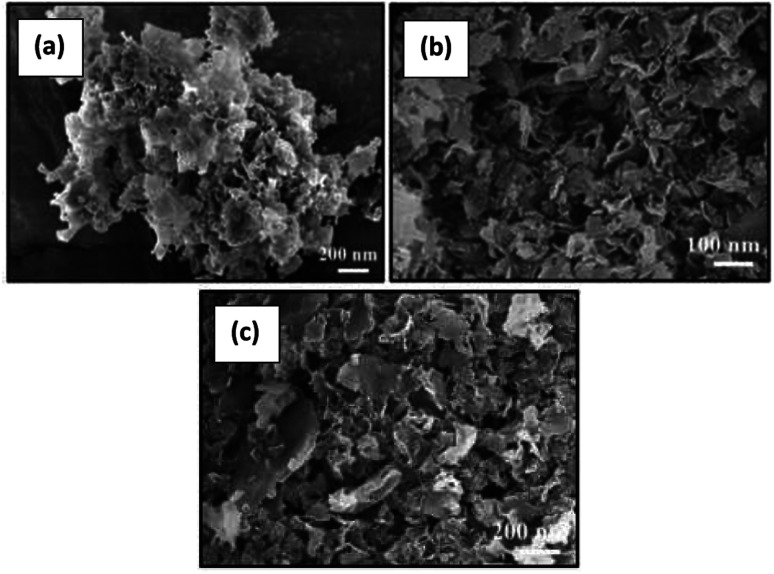
FE-SEM images of (a) g-C_3_N_4_ (b) 2 NZCN (c) 2 SZCN nanocomposite.

The Brunauer–Emmett–Teller (BET) specific surface areas of the photocatalysts were evaluated by their N_2_ adsorption–desorption isotherms (Fig. S3(a)–(c)†). The BET specific surface area of pure g-C_3_N_4_ and N-doped ZnO and S-doped ZnO nanoparticles were obtained as 66.5 m^2^ g^−1^, 147.9 m^2^ g^−1^ and 117.4 m^2^ g^−1^, respectively. The specific surface areas of the 2 NZCN and 2 SZCN nanocomposites are 89 m^2^ g^−1^ and 84 m^2^ g^−1^, respectively, which are lower than those of the pure N-doped ZnO and S-doped ZnO nanoparticles due to filling the interparticle space of the parent g-C_3_N_4_ photocatalyst with N-doped ZnO and S-doped ZnO NPs, respectively (Fig. S4(a) and (b)†). Table S1† summarizes the BET surface areas of the as-synthesized N-doped ZnO@g-C_3_N_4_ and S-doped ZnO@g-C_3_N_4_ nanocomposites with different contents of N-doped ZnO and S-doped ZnO nanoparticles, respectively. As can be seen, with an increase in the contents of N-doped ZnO and S-doped ZnO nanoparticles, there is a gradual decrease in the specific surface area values in the nanocomposites. Low contents of N-doped ZnO and S-doped ZnO NPs with larger surface areas results in lower catalytic efficiencies than those of the 2 NZCN and 2 SZCN nanocomposites, as they can produce few electron–hole pairs for the photocatalytic process. However, with an increase in the content of N-doped ZnO and S-doped ZnO up to 6 wt%, the catalytic activity deceases because the low surface area restricts the contact between pollutant and photocatalyst. A morphological evaluation of the as-synthesized photocatalysts was conducted by FESEM, TEM and HRTEM analysis. Fig. 3(a)–(c) show the FESEM images of bare g-C_3_N_4_ and N-doped ZnO@g-C_3_N_4_ and S-doped ZnO@g-C_3_N_4_ nanocomposites. As depicted in Fig. 3(a), bare g-C_3_N_4_ consists of lamellar shaped 2D nanosheets with a smooth surface. In Fig. 3(b) and (c), g-C_3_N_4_ retains its flat layer structure even after the loading of N-doped ZnO and S-doped ZnO nanoparticles, respectively.

**Fig. 4:**
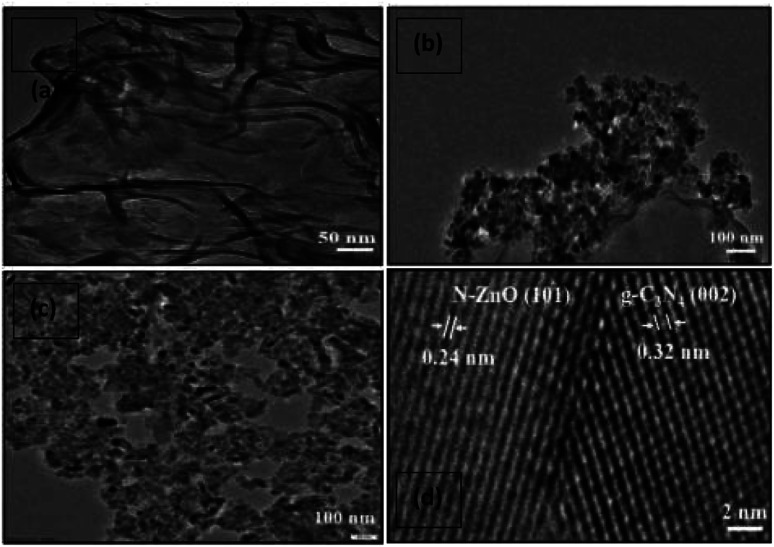
TEM images of (a) g-C_3_N_4_, (b) 2 NZCN, and (c) 2 SZCN. (d) HRTEM image of 2 NZCN nanocomposite.

The small, aggregated structures of the N-doped ZnO and S-doped ZnO nanoparticles are clearly embedded over the g-C_3_N_4_ nanosheets and thus lead to the construction of 2D–2D nanocomposites between g-C_3_N_4_ and the N-doped ZnO and S-doped ZnO nanoparticles (Fig. 4(b) and (c)).

To investigate the microstructure and morphology of the photocatalysts, TEM and HRTEM were performed. As shown in Fig. 4(a), bare g-C_3_N_4_ exhibits planar, thin, stacked layers and an irregular sheet-like morphology. In Fig. 4(d), the lattice plane of 0.24 nm corresponds to the (101) diffraction plane of ZnO and the lattice plane of 0.32 nm corresponds to the characteristic (002) plane of g-C_3_N_4_ in the N-doped ZnO@g-C_3_N_4_ nanocomposite. Therefore, the interfacial contact of the N-doped ZnO (101) with the g-C_3_N_4_ (002) lattice plane inhibits the recombination probability, hence improving the catalytic activity.^13^

The EDX elemental mapping analysis clearly reveals the presence of the constituent elements (C, N, O and Zn) in the 2 wt% N-doped ZnO@g-C_3_N_4_ nanocomposite, as depicted in Fig. S5.† Similarly, the EDX elemental mapping analysis of the 2 wt% S-doped ZnO@g-C_3_N_4_ nanocomposite indicates the presence of C, N, O, Zn and S elements, as depicted in Fig. S6.†

**Fig. 5:**
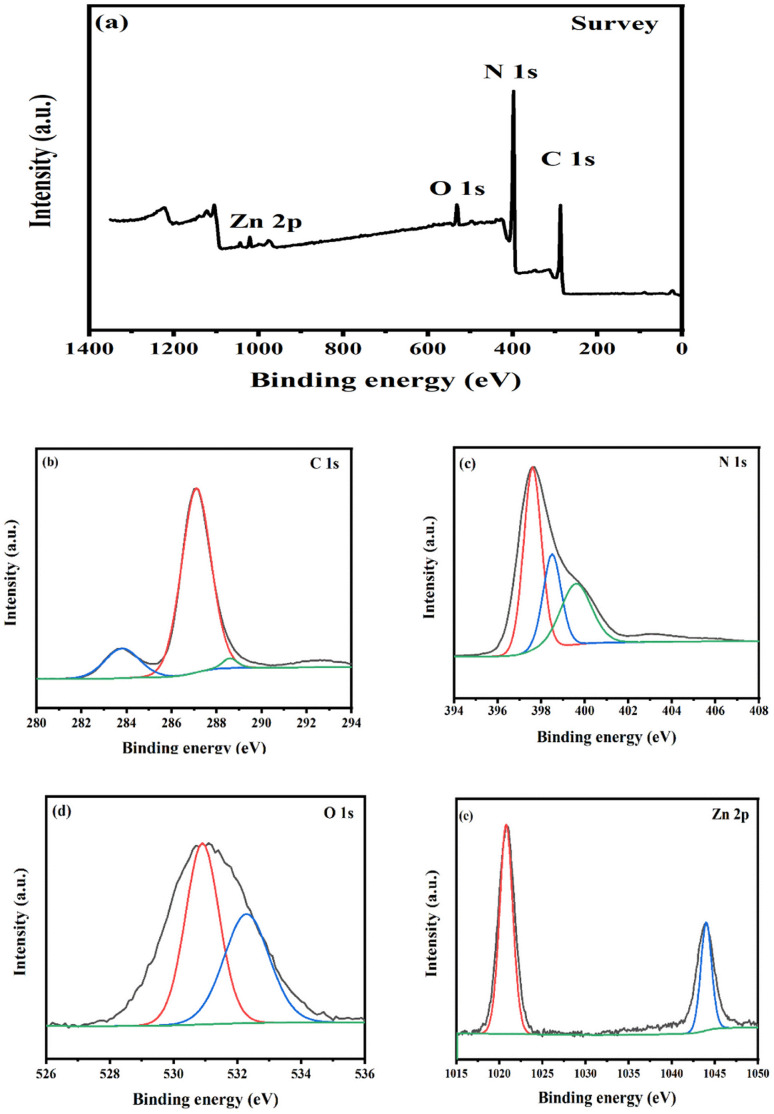
(a) XPS survey spectrum, (b) C 1s XPS spectrum, (c) N 1s XPS spectrum, (d) O 1s XPS spectrum and (e) Zn 2p XPS spectrum of 2 NZCN nanocomposite.

**Fig. 6:**
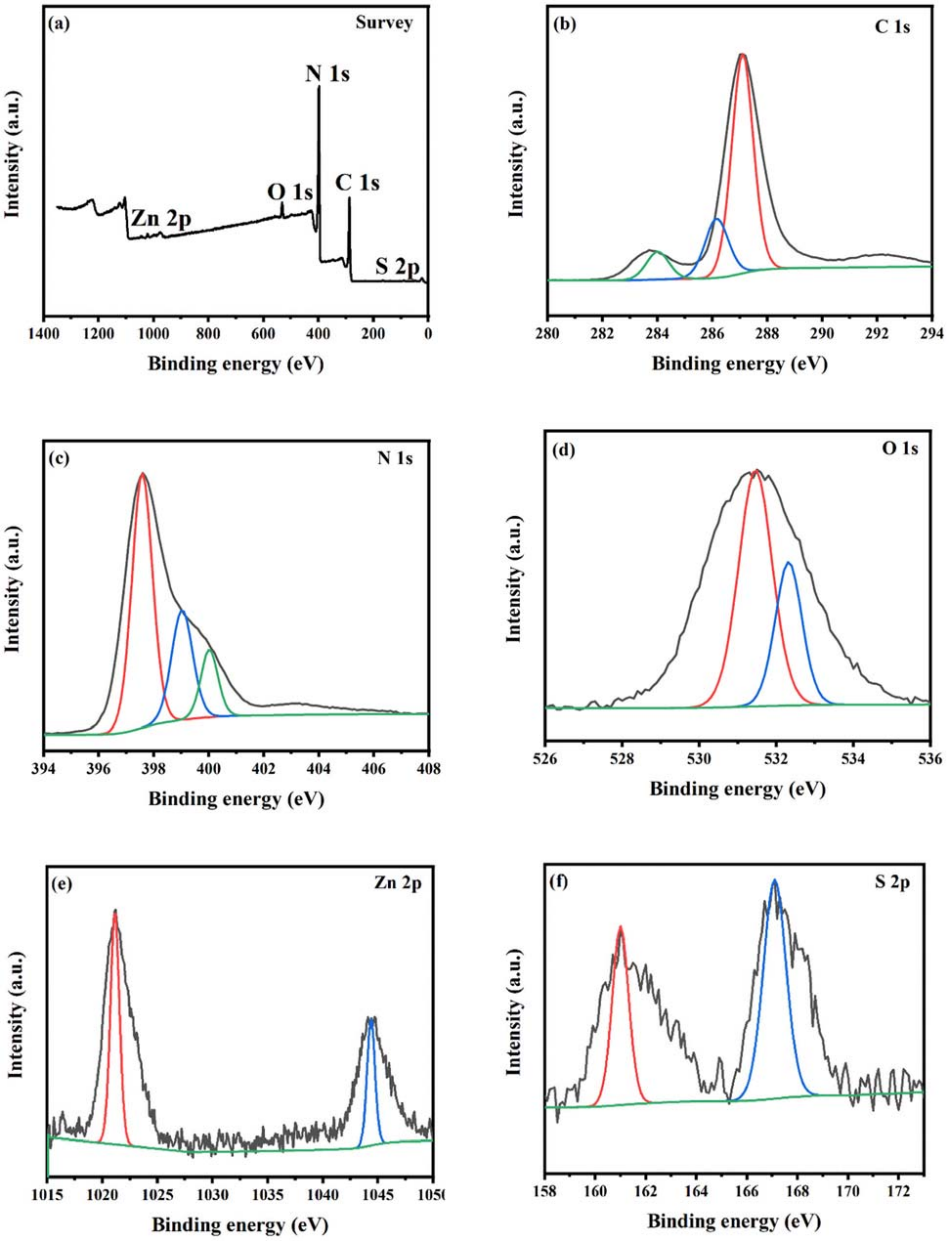
(a) XPS survey spectrum, (b) C 1s XPS spectrum, (c) N 1s XPS spectrum, (d) O 1s XPS spectrum, (e) Zn 2p XPS spectrum and (f) S 2p XPS spectrum of 2 SZCN nanocomposite.

XPS analysis was performed in order to explicate the surface chemical composition and the chemical state of the photocatalyst. As depicted in Fig. 5(a) and 6(a), the survey XPS of N-doped ZnO@g-C_3_N_4_ and S-doped ZnO@g-C_3_N_4_ nanocomposites reveal the presence of C 1s, N 1s, O 1s, and Zn 2p elements and C 1s, N 1s, O 1s, Zn 2p, and S 2p elements, respectively. Regarding C 1s in Fig. 5(b) and 6(b), the three major peaks at 283.79 eV, 287.08 eV and 288.65 eV in 2 NZCN and the peaks at 284.05 eV, 286.17 eV and 287.08 eV in the 2 SZCN nanocomposite are associated with sp^2^ C–C bonds, sp^3^ coordinated carbon species from the defects on the g-C_3_N_4_ surface and C–N–C coordination, respectively.^29,31^ The high-resolution spectra of N 1s display three peaks at binding energies of 397.59 eV, 398.50 eV and 399.69 eV in the 2 NZCN nanocomposite and 397.62 eV, 399.01 eV and 400.06 eV in the 2 SZCN nanocomposite (Fig. 5(c) and 6(c)). The binding energies at 397.59 eV and 397.62 eV may be attributed to sp^2^ bonded carbon in nitrogen-containing aromatic rings (C=N–C) and the two peaks at 399.69 eV and 399.01 eV and the one at 400.06 originate from N-(C)_3_ units and the N atom in amino moieties, respectively.^31,32^ In the N 1s spectrum of 2 NZCN nanocomposite, the N 1s peak at 398.50 eV corresponds to the Zn–N nitrogen, which clearly shows the successful doping of N at the O sites of ZnO.^8^ In Fig. 5(d) and 6(d), the high-resolution spectrum of O 1s can be dissociated into two major peaks at 530.92 eV and 532.34 eV for the 2 NZCN nanocomposite and at 531.49 eV and 532.32 eV for the 2 SZCN nanocomposite, corresponding to the lattice oxygen of ZnO and absorbed water on the catalyst surface, respectively.^26^ Peaks at 1020.79 eV and 1044 eV and 1021.15 eV and 1044.43 eV are observed in the high-resolution spectra of Zn 2p in 2 NZCN and 2 SZCN nanocomposites, respectively (Fig. 5(e) and 6(e)), and correspond to Zn 2p_3/2_ and Zn 2p_1/2_, respectively.^26,29^ As shown in Fig. 6(f), the high-resolution spectrum of S 2p could be dissociated into two major peaks at 160.99 eV and 167.08 eV, ascribed to the C–S bond and sulphur oxide powder during calcination process, respectively, which evidences the successful doping of S into the crystalline ZnO substrate.^26,33^

**Fig. 7:**
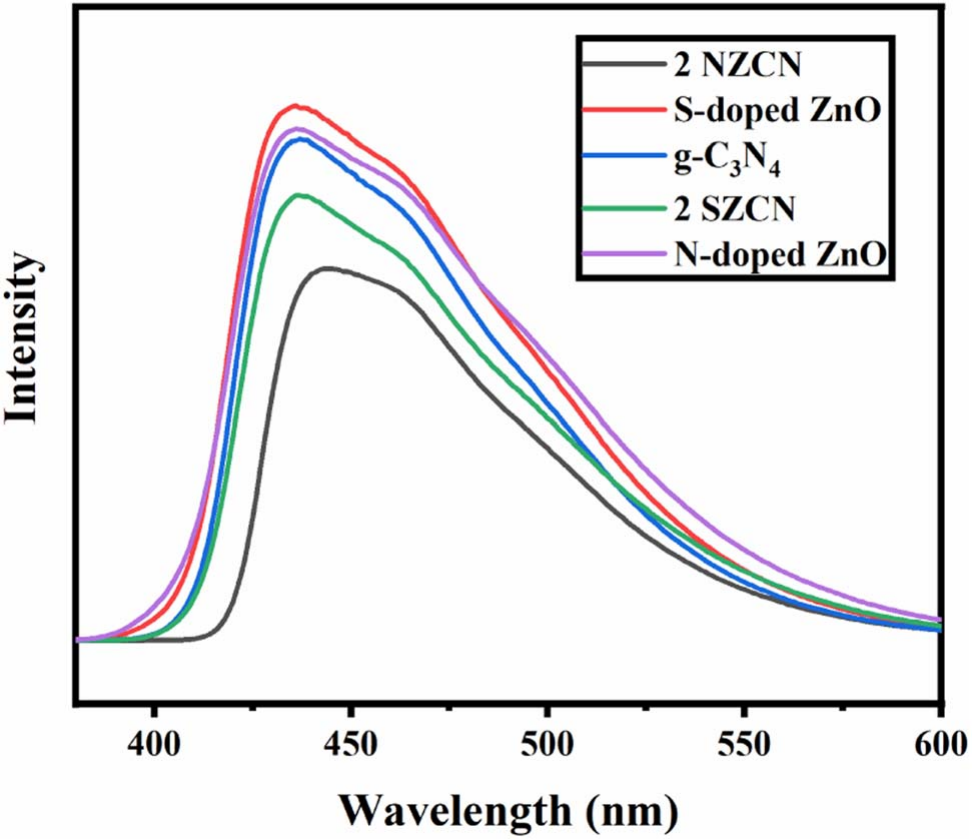
Photoluminescence spectra of g-C_3_N_4_, N-doped ZnO, S-doped ZnO, and 2 NZCN and 2 SZCN nanocomposites.

The room temperature PL spectra of the as-synthesized catalysts were studied to evaluate the separation and recombination probability of charge carriers in g-C_3_N_4_ and the N-doped ZnO and S-doped ZnO nanoparticles at 325 nm (Fig. 7). As depicted in Fig. 7, the bare g-C_3_N_4_ has a major emission peak centered at about 425 nm indicating the band–band PL phenomenon.^34^ Remarkably, the PL emission intensity becomes weaker for the N-doped ZnO@ g-C_3_N_4_ and S-doped ZnO@g-C_3_N_4_ nanocomposites with the addition of the N-doped ZnO and S-doped ZnO nanoparticles, respectively, to the g-C_3_N_4_ nanosheets. The PL emission intensity increases due to the recombination probability of charge carriers, and the decrease in the emission intensity indicates the suppressed recombination of charge carriers.^35^ Therefore, N-doped ZnO@ g-C_3_N_4_ and S-doped ZnO@g-C_3_N_4_ nanocomposites more effectively inhibit the recombination of photo-excited charge carriers for better photocatalytic activity.

**Fig. 8:**
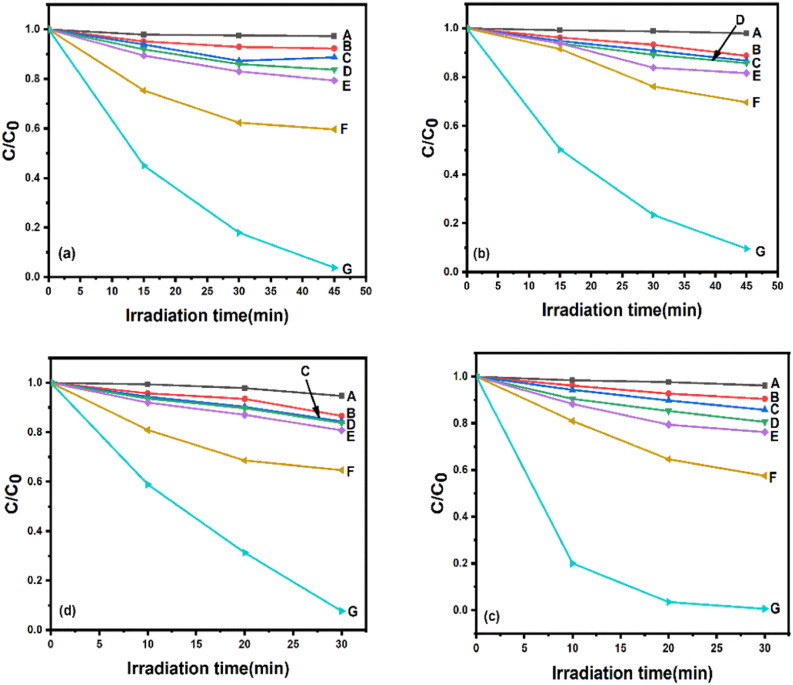
Photocatalytic degradation of (a) CV by 2 NZCN nanocomposite, (b) CV by 2 SZCN nanocomposite, (c) BG by 2 NZCN nanocomposite, and (d) BG by 2 SZCN nanocomposite under various conditions: (A) blank, (B) H_2_O_2_, (C) H_2_O_2_/vis, (D) Cat (E) Cat/vis, (F) Cat/H_2_O_2_, (G) Cat/H_2_O_2_/vis.

The photocatalytic activities of N-doped ZnO@g-C_3_N_4_ and S-doped ZnO@g-C_3_N_4_ nanocomposites were evaluated in terms of the visible-light assisted degradation of two dye molecules, namely crystal violet (CV) and brilliant green (BG). UV-vis analysis visibly indicates the changes in the molecular structures of CV and BG in visible-light-driven photodegradation using the 2 NZCN and 2 SZCN photocatalysts. The characteristic absorption bands of CV and BG, located at 590 and 625 nm, respectively,^36,37^ decrease rapidly as the irradiation time increases and nearly vanish after 30 min and 45 min of visible-light assisted irradiation by the 2 NZCN and 2 SZCN photocatalysts, respectively. As depicted in Fig. 8(a)–(d), the discolorations of CV and BG, respectively, follow the order Cat/H_2_O_2_/vis > Cat/vis > Cat/H_2_O_2_ > Cat > H_2_O_2_/vis > H_2_O_2_. In the blank experiment, the direct photolysis of CV and BG is almost negligible without the photocatalyst, indicating that the degradation of CV and BG arises from the photocatalytic reaction instead of photolysis. The discoloration due to the photocatalyst hardly occurred in the absence of H_2_O_2_ and visible light, suggesting that the photocatalyst did not lead to significant adsorption of the dyes. In the presence of the catalyst, visible light and H_2_O_2_, the discoloration of dye was significantly enhanced, suggesting the crucial role of the as-synthesized N-doped ZnO@g-C_3_N_4_ and S-doped ZnO@g-C_3_N_4_ nanocomposites towards the efficient degradation of both CV and BG dyes. The as-synthesized 2 NZCN nanocomposite exhibits the highest catalytic efficiency towards degradation of the dyes, *i.e.* 96.2% of CV is degraded within 45 min and 99.3% of BG is degraded within 30 min of H_2_O_2_ assisted visible-light irradiation by 2 NZCN photocatalyst. Similarly, in the presence of both visible light and H_2_O_2_, the as-prepared 2 SZCN photocatalyst exhibits 90.4% CV degradation and 92.3% BG degradation.

The consumption and decomposition of H_2_O_2_ in the photocatalytic degradation of CV and BG by the as-synthesized 2 NZCN and 2 SZCN photocatalysts under various conditions were evaluated by a simple and fast spectrophotometric metavanadate assisted method^38^ and followed the order Cat/H_2_O_2_/vis > Cat/H_2_O_2_ > H_2_O_2_/vis > H_2_O_2_. As seen in Fig. S7(a) and (c),† it was observed that, in the presence of 2 NZCN photocatalyst and visible light, the highest consumption of H_2_O_2_ was observed as 97% and 96.6% for CV and BG degradations, respectively. Similarly, in H_2_O_2_ assisted CV and BG degradation by 2 SZCN photocatalyst, the consumption of H_2_O_2_ was found to be 96.4% and 97.3%, as shown in Fig. S7(b) and (d),† respectively.

**Fig. 9:**
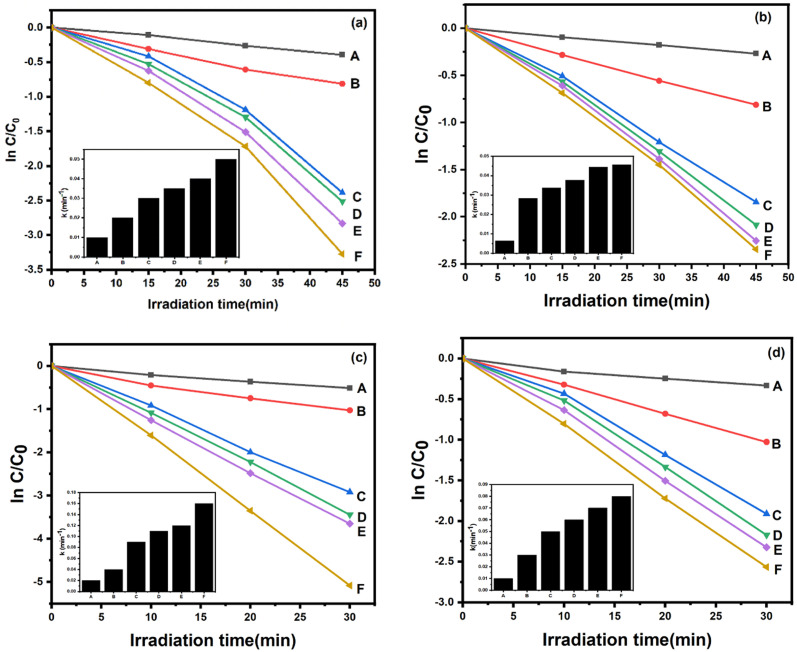
Kinetics of CV degradation over different catalysts. (a and c) (A) N-doped ZnO NPs, (B) g-C_3_N_4_, (C) 6 wt% N–ZnO@g-C_3_N_4_, (D) 4 wt% N–ZnO@g-C_3_N_4_, (E) 1 wt% N–ZnO@g-C_3_N_4_, (F) 2 wt% N–ZnO@g-C_3_N_4_. (b and d) (A) S-doped ZnO NPs, (B) g-C_3_N_4_, (C) 6 wt% S–ZnO@g-C_3_N_4_, (D) 4 wt% S–ZnO@g-C_3_N_4_, (E) 1 wt% S–ZnO@g-C_3_N_4_, (F) 2 wt% S–ZnO@g-C_3_N_4_. Inset: degradation rate constants *k*. (Reaction conditions: [dye] = 100 mg L^−1^, catalyst = 200 mg L^−1^, *T* = 25 °C, pH = 6, [H_2_O_2_] = 0.10 M).

The photocatalytic efficiencies of the as-prepared N-doped ZnO@g-C_3_N_4_ and S-doped ZnO@g-C_3_N_4_ nanocomposites were examined in the visible-light assisted photodegradation of CV and BG. The catalytic efficiencies of the N-doped ZnO and S-doped ZnO are higher compared to that of bare ZnO, which clearly signifies that the N- and S-doping considerably enhance the spectral response to the visible region (Fig. 9(a), (c) and (b), (d)). The g-C_3_N_4_, pure N-doped ZnO and pure S-doped ZnO show low photocatalytic degradation efficiencies of almost 55.6%, 32.6% and 23.6%, respectively, in the photocatalytic degradation of CV. Similarly, the photocatalytic efficiencies of g-C_3_N_4_, pure N-doped ZnO and pure S-doped ZnO are found to be 64.2%, 40.2% and 28.3%, respectively, for the degradation of BG. After being combined with g-C_3_N_4_, the catalytic efficiencies of N-doped ZnO@g-C_3_N_4_ and S-doped ZnO@g-C_3_N_4_ nanocomposites improved significantly and the weight percentages of pure N-doped ZnO and S-doped ZnO have significant influence on the degradation efficiencies. The catalysts with 2 wt% N-doped ZnO and 2 wt% S-doped ZnO loading exhibit the best photocatalytic performance in dye degradation, resulting in visible-light induced photocatalytic activities of 96.2% and 90.4% within 45 min for CV degradation and 99.3% and 92.3% within 30 min for BG degradation, respectively. The ICP-MS analysis indicated that the Zn contents in 2 NZCN and 2 SZCN were found to be 9.0 ppm and 13.37 ppm, respectively. Similarly, the catalyst turnover numbers (TON) for the 2 NZCN supported photocatalytic degradations of BG and CV were calculated on the basis of Zn content to be 1.7 × 10^4^ and 1.1 × 10^4^, respectively. Accordingly, for the 2 SZCN catalyzed photocatalytic degradation of BG and CV, the catalyst turnover numbers (TON) were found to be 1.1 × 10^4^ and 6.7 × 10^3^, respectively, based on Zn content, indicating higher catalytic activity towards efficient degradation. Therefore, developing novel catalysts for reactions with high TONs is advantageous for practical applications.^39^

The reaction processes of CV and BG photodegradation over g-C_3_N_4_, pure N-doped ZnO and pure S-doped ZnO with different loadings of N-doped ZnO and S-doped ZnO successfully follow the pseudo-first-order chemical kinetic model in eqn (2).

2 ln(*C*/*C*_0_) = −*kt*

In this equation, *k* represents the first-order rate constant (min^−1^), *C*_0_ is the initial concentrations of CV and BG and *C* represents the concentrations at reaction time *t*. The *k* values of all the photocatalysts for CV and BG degradation were calculated and are depicted in the insets of Fig. 9(a), (c) and (b), (d), respectively. The as-synthesized 2% N-doped ZnO@g-C_3_N_4_ and 2% S-doped ZnO@g-C_3_N_4_ nanocomposites exhibited rate constants of 0.05 min^−1^ and 0.04 min^−1^ for CV and 0.16 and 0.08 for BG, respectively. The rate constants of 2 NZCN are 2.5 and 5 times higher than those of g-C_3_N_4_ and N-doped ZnO nanoparticles, respectively, in the case of CV. Similarly, the rate constants of 2 SZCN are 2 and 6.6 times higher than those of g-C_3_N_4_ and S-doped ZnO nanoparticles, respectively, in the case of CV. A significant enhancement of the photocatalytic efficiency of 2 NZCN nanocomposite towards the degradation of BG was seen, at 5.3 and 16 times higher than those of g-C_3_N_4_ and N-doped ZnO nanoparticles, respectively. For the photodegradation of BG, the optimal catalytic efficiency of 2 SZCN nanocomposite is 2.6 and 8 times higher than those of g-C_3_N_4_ and S-doped ZnO nanoparticles, respectively. However, with further increases in the contents of N-doped ZnO and S-doped ZnO for the N-doped ZnO@g-C_3_N_4_ and S-doped ZnO@g-C_3_N_4_ nanocomposites, the degradation efficiencies decrease due to the excessive amount of N-doped ZnO and S-doped ZnO on the g-C_3_N_4_ surface leading to the reduction of heterojunctions between the g-C_3_N_4_ and the N-doped ZnO and S-doped ZnO. Furthermore, the number of active sites on g-C_3_N_4_ was reduced, which is necessary to quench the holes due to the excessive amounts of N-doped ZnO and S-doped ZnO.^40^

The role of initial H_2_O_2_ concentration on the visible-light assisted degradation efficiencies for CV and BG can be considered a major factor. In the case of photocatalytic degradation of CV by the 2 NZCN and 2 SZCN nanocomposites, it was observed that, with increasing initial H_2_O_2_ concentration from 0.005 M to 0.10 M, the pseudo first order kinetic constant (*k*) increased gradually from 0.01 to 0.05 and 0.02 to 0.04, respectively, and was then saturated beyond 0.01 M (Fig. S8(a) and (b)†). Similarly, the rate constant value enhanced considerably from 0.02 to 0.16 and 0.03 to 0.08 in the concentration range 0.005 M to 0.10 M for the degradation of BG by 2 NZCN and 2 SZCN nanocomposites, respectively, as shown in Fig. S9(a) and (b).† This result shows that only a small amount of H_2_O_2_ is required to efficiently run the photocatalytic reactions. The excessive amount of H_2_O_2_ essentially acts as a self-scavenger for ˙OH according to eqn (3) and (4).^41^

3 H_2_O_2_ + ˙OH → ˙OOH + H_2_O

4 ˙OOH + ˙OH → H_2_O + O_2_

The pH effect may be considered one of the most impactful parameters. The influence of pH on the catalytic process for both CV and BG by our as-synthesized 2 NZCN and 2 SZCN photocatalysts is illustrated in Fig. S10(a)–(d),† respectively. The effects of initial pH on the photodegradation of both CV and BG were investigated by changing the pH of the dye solution from 2 to 11, keeping the catalyst dose (200 mg L^−1^) and concentration of dye (100 ppm) constant. The pH of the suspension was varied by adding the proper quantity of NaOH or HCl. The photocatalytic degradation rates of both cationic dyes CV and BG increase with a gradual increase in pH value and finally achieve the maximum rate at pH = 9. Thereafter, further enhancement of the pH leads to a decrease in catalytic activity because the higher pH leads the semiconductor surface to become negatively charged, which is favourable for better adsorption of the cationic dye molecule onto the catalyst surface.^42^ Also, a higher pH value leads to the production of more of active species ˙OH due to the interaction between the photo-excited holes (h^+^) of the catalyst and the OH^−^ of the base.^43^ This type of close interaction of the adsorbed dye molecule with ˙OH radicals subsequently causes the higher rate of catalytic activity. However, at pH > 9, the cationic dye changes into a neutral molecule, thereby making adsorption onto the photocatalyst surface difficult and resulting in a decrease in degradation efficiency.

**Fig. 10:**
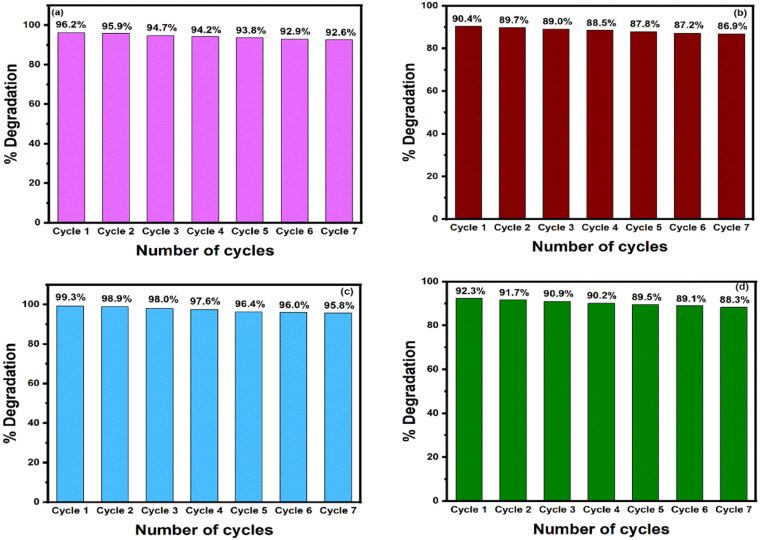
Reusability studies of (a) 2 NZCN nanocomposite for the photodegradation of CV, (b) 2 SZCN nanocomposite for the degradation of CV, (c) 2 NZCN nanocomposite for the degradation of BG, and (d) 2 SZCN nanocomposite for the degradation of BG under visible-light irradiation. (Reaction conditions: [dye] = 100 mg L^−1^, catalyst = 200 mg L^−1^, *T* = 25 °C, pH = 6, [H_2_O_2_] = 0.10 M).

The stability and recyclability of photocatalysts are considered very important factors in photocatalytic reactions. To evaluate the stability and recyclability of the as-synthesized 2 NZCN and 2 SZCN nanocomposites, seven successive photocatalytic experimental runs were carried out with the addition of recycled 2 NZCN and 2 SZCN nanocomposites to fresh CV and BG solutions at the same concentration of the catalyst under visible-light irradiation. Prior to the reusability experiments, the catalyst was filtered and dried at 100 °C. The catalytic activity of 2 NZCN nanocomposite for the degradation of CV and BG remains almost consistent, decreasing only from 96.2% to 92.6% in the case of CV and from 99.3% to 95.8% in the case of BG even after seven successive runs, indicating the good recyclability of the photocatalyst (Fig. 10(a) and (c)). Similarly, the photocatalytic activity of 2 SZCN nanocomposite was retained, from 90.4% to 86.9% (in the case of CV solution) and from 92.3% to 88.3% (in the case of BG solution) after the seventh catalytic cycle, indicating the photostability of the catalysts for potential applications (Fig. 10(b) and (d)). Therefore, N-doped ZnO@g-C_3_N_4_ and S-doped ZnO@gC_3_N_4_ nanocomposites act as stable photocatalytic materials towards environmental protection. Furthermore, there is no obvious difference in the SEM images of 2 NZCN and 2 SZCN nanocomposites, as depicted in Fig. S11(a) and (b),† respectively, after the seventh catalytic cycle. XPS analysis of the recovered 2 NZCN and 2 SZCN photocatalysts (Fig. S12 and S13†) indicates their high chemical stability in the photodegradation process. Therefore, N-doped ZnO@g-C_3_N_4_ and S-doped ZnO@g-C_3_N_4_ nanocomposites act as stable photocatalytic materials towards environmental protection.

**Fig. 11:**
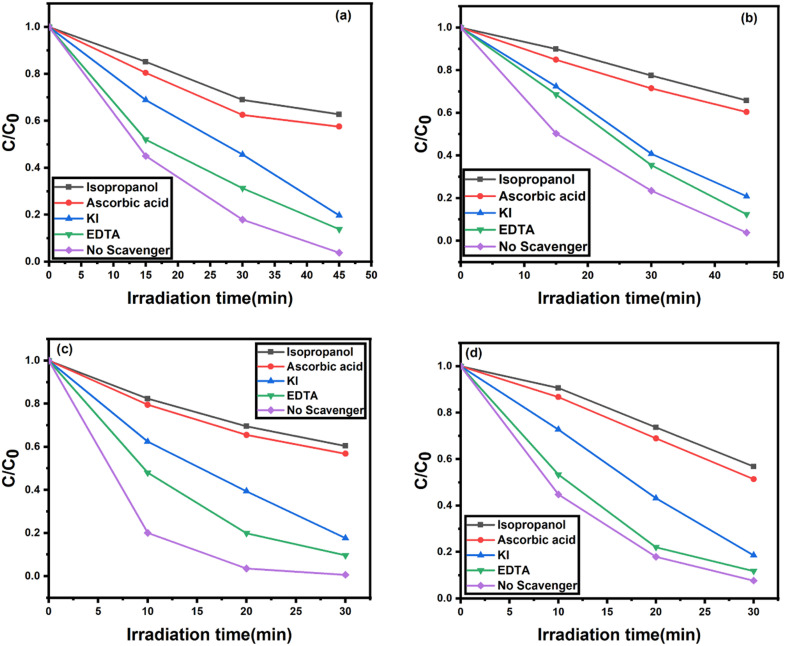
Trapping experiments of active species on the photodegradation of (a) CV by 2 NZCN nanocomposite, (b) CV by 2 SZCN nanocomposite, (c) BG by 2 NZCN nanocomposite, and (d) BG by 2 SZCN nanocomposite. (Reaction conditions: [dye] = 100 mg L^−1^, catalyst = 200 mg L^−1^, *T* = 25 °C, pH = 6, [H_2_O_2_] = 0.10 M).

To investigate the photocatalytic degradation mechanism, various scavengers were employed to investigate the major oxidative species involved in the photodegradation reaction. Here, isopropyl alcohol, ascorbic acid and EDTA were utilized as ˙OH scavenger, ˙O_2_^−^ scavenger and h^+^ scavenger, respectively, while potassium iodide was applied to reduce both ˙OH and h^+^ radical. Fig. 11(a) and (c) depict the photocatalytic efficiencies of 2 NZCN nanocomposite with CV and BG, respectively, in the presence of various scavengers. When IPA was added into the reaction mixture, the photocatalytic efficiencies were significantly reduced to 37.2% and 39.6%, respectively, indicating the major role of ˙OH radical in the degradation mechanism of CV and BG by 2 NZCN photocatalyst. Similarly, after the addition of ascorbic acid, the photocatalytic efficiencies are reduced to 42.4% and 43.2% for CV and BG, as depicted in Fig. 11(a) and (c), respectively, indicating the major role of the ˙O_2_^−^ radical in the photocatalytic process. However, the photocatalytic efficiencies were almost unvarying with the addition of EDTA, suggesting the hole is not the major active species. For the 2 SZCN photocatalyst, upon the addition of IPA, the conversion percentages of CV and BG were greatly decreased to 34.2% and 43.2%, respectively. Similarly, the addition of ascorbic acid decreases the photocatalytic efficiencies of both CV and BG to 39.6% and 48.6%, respectively (Fig. 11(b) and (d)). The addition of EDTA led to non-significant changes, indicating that h^+^ plays a negligible role in the photodegradation of CV and BG over both the 2 NZCN and 2 SZCN photocatalysts.

The positions of the valence band and conduction band of the as-synthesized photocatalysts could be found by using eqn (5) and (6).^44^

5 *E*_VB_ = *χ* − *E*_e_ + 0.5*E*_bg_

6 *E*_CB_ = *E*_VB_ − *E*_bg_

In the above equations, *E*_VB_, *E*_CB_, *χ*, *E*_e_ and *E*_bg_ denote the valence band edge potential, the conduction band edge potential, the electronegativity of the semiconductor, the energy of the free electrons on the hydrogen scale, *i.e.* 4.5, and the band gap energies of g-C_3_N_4_, N-doped ZnO and S-doped ZnO, *i.e.* 2.77 eV, 2.99 eV and 3.05 eV, respectively. Hence, the *E*_CB_ values of g-C_3_N_4_, N-doped ZnO and S-doped ZnO are obtained as −1.24 eV, −0.20 eV and −0.23 eV, respectively. The *E*_VB_ values of g-C_3_N_4_, N-doped ZnO and S-doped ZnO are determined to be 1.53 eV, 2.79 eV and 2.82 eV, respectively.

**Fig. 12:**
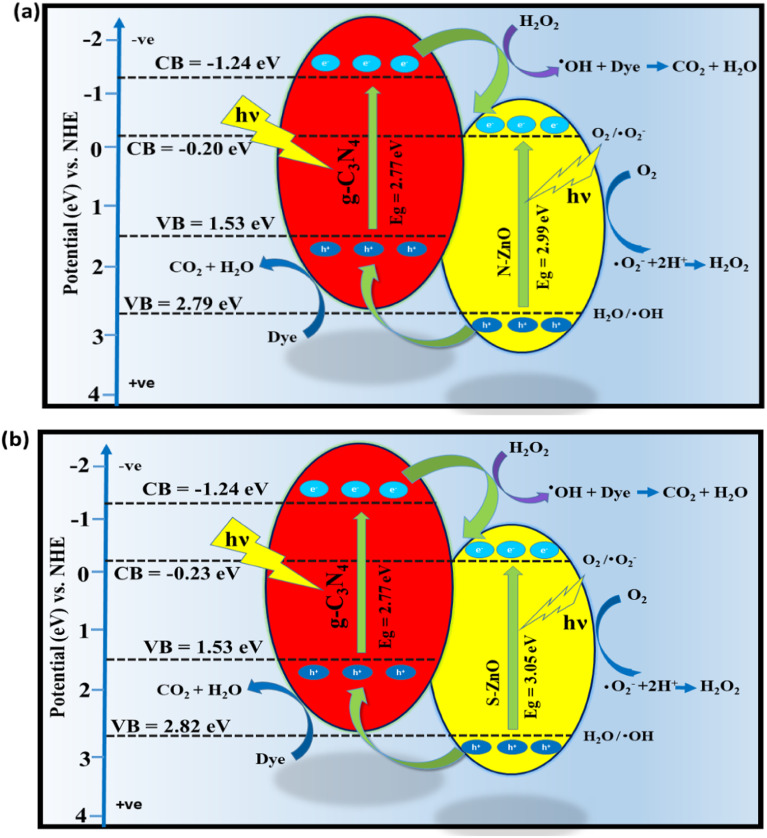
Proposed mechanism for the photodegradation of CV and BG by (a) N-doped ZnO@g-C_3_N_4_ nanocomposite and (b) S-doped ZnO@g-C_3_N_4_ nanocomposite under visible-light irradiation.

Generally, semiconductor-based photocatalytic reactions are initiated by the generation of some photo-excited charge carriers. A plausible mechanism for the visible-light assisted photocatalytic degradation of both CV and BG by the as-synthesized N-doped ZnO@g-C_3_N_4_ and S-doped ZnO@g-C_3_N_4_ nanocomposites is illustrated in Fig. 12(a) and (b), respectively. In the presence of a visible-light source, the movement of photo-excited charge carriers in g-C_3_N_4_, N-doped ZnO and S-doped ZnO from the VB to the CB separate the photo-generated charge carriers on the photocatalyst surface. The photo-excited electrons could easily migrate from the CB of g-C_3_N_4_ to the CB of N-doped ZnO and S-doped ZnO, as the CB level of g-C_3_N_4_ is more negative than those of N-doped ZnO and S-doped ZnO, thereby enhancing the separation of the photo-excited charge carriers.^9^ In both N-doped ZnO and S-doped ZnO, the photo-generated electron in the CB can react with dissolved O_2_ to produce more active radicals which promote the dye degradation.^45^ The photo-induced electrons lead to the formation of more active radical superoxide anions (˙O_2_^−^) for the photocatalytic process. However, the majority of photo-excited holes on the VB of N-doped ZnO migrated to the VB of g-C_3_N_4_, as the VB potential of g-C_3_N_4_ (+1.4 eV NHE) is less positive than the standard redox potential *E*^0^ (H_2_O/˙OH) (+2.4 eV *vs.* NHE).^46^ Hence, the photo-excited holes can lead to the formation of ˙OH radicals for complete mineralization of the organic dye molecules.^45^ However, these ˙OH radicals can also be trapped using salicylic acid as a trapping agent.^47^ Compared with the individual components, the formation of N-doped ZnO@g-C_3_N_4_ and S-doped ZnO@g-C_3_N_4_ heterojunctions induces higher photocatalytic activity towards the degradation of organic pollutants.

A comparison of some previously reported catalysts with the present catalysts towards the degradation of both crystal violet and brilliant green dyes is summarized in Table S2.†

## Conclusions

In summary, visible-light-driven N-doped ZnO@g-C_3_N_4_ and S-doped ZnO@g-C_3_N_4_ photocatalysts were prepared by following a simple, cost-effective sonication assisted route. Compared to the bare catalysts, the as-synthesized 2 wt% N-doped ZnO@g-C_3_N_4_ and 2 wt% S-doped ZnO@g-C_3_N_4_ nanocomposites exhibited higher catalytic efficiency towards the visible-light assisted degradation of CV and BG. The as-synthesized 2 NZCN photocatalyst showed 2.5 and 5 times higher photocatalytic efficiency compared to bare g-C_3_N_4_ and N-doped ZnO, respectively, towards the degradation of CV and 5.3 and 16 times higher efficiencies compared to pure g-C_3_N_4_ and N-doped ZnO, respectively. Similarly, the visible-light assisted photodegradation of CV by 2 SZCN exhibited 2 and 6.6 times higher photocatalytic efficiencies, with 2.6 and 8 times greater catalytic efficiencies in the case of BG, compared to g-C_3_N_4_ and S-doped ZnO, respectively. In the photocatalytic processes of both CV and BG, ˙O_2_^−^ and ˙OH are the major active species. The photocatalyst also exhibited excellent stability and efficiency even after seven catalytic cycles. The highly enhanced photocatalytic performance and excellent stability of the as-synthesized photocatalysts may be ascribed to the effective charge transfer of the photo-excited species at the heterojunction interfaces and increased light absorbance. This research offers a facile and cost effective route for the synthesis of photocatalysts with excellent catalytic efficiency towards visible-light assisted degradation of environmental pollutants.

## Experimental

All reagents and chemicals were analytical grade, purchased from Sigma-Aldrich company sources, and used as received. Distilled water was used throughout.

Typically, 20 g of urea was placed in a well-closed silica crucible and heated at 4 °C min^−1^ to 550 °C for 2 h in a muffle furnace. The as-synthesized g-C_3_N_4_ was collected and ground into powder. The g-C_3_N_4_ nanosheets were synthesized by exfoliating the bulk g-C_3_N_4_ in deionized water. 200 mg of the obtained g-C_3_N_4_ was dispersed in 100 mL of distilled water, followed by ultrasonication and centrifugation at 15 000 rpm.

In a typical process, zinc acetate dihydrate (0.10 mol) was put into 30 mL of distilled water and the suspension was stirred for 30 min in order to get a clear solution, followed by heating above 100 °C to get our desired pure ZnO nanoparticles.^27^

N-doped ZnO nanoparticles were prepared based on the previously reported method with a little modification.^27^ In a typical synthesis, zinc acetate dihydrate was mixed thoroughly with urea (molar ratio 1 : 2) using a mortar and pestle, followed by adding 20 mL of distilled water. The colourless solution of zinc acetate urea complex was stirred, followed by heating at 100 °C in order to evaporate the water. The as-synthesized pale yellow coloured gel was washed, dried at 70 °C for 48 h and heated at 500 °C for 3 h to get N-doped ZnO nanoparticles.

The S-doped ZnO nanoparticles were prepared according to the following procedure. Typically, zinc acetate dihydrate and sulphur powder were mixed at a molar ratio of 1 : 2. The resulting mixture was ultrasonically dispersed in 200 mL of water for 1 h in order to get a yellowish coloured solution. The suspension was dried using a Rotavapor followed by oven drying at 100 °C overnight. The dried product was calcined at 500 °C for 3 h in a muffle furnace.

An appropriate quantity of g-C_3_N_4_ was exfoliated in 100 mL of distilled water for 30 min. Different amounts of as-synthesized N-doped ZnO (1, 2, 4 and 6 mg) or S-doped ZnO (1, 2, 4 and 6 mg) were added into the suspension and ultrasonicated overnight. In order to get the desired N-doped ZnO@g-C_3_N_4_ and S-doped ZnO@g-C_3_N_4_ nanocomposites, water was removed with the help of a Rotavapor, followed by oven drying at 100 °C for 1 h. To investigate the effect of the loadings of N-doped ZnO and S-doped ZnO in the photocatalytic study, the corresponding samples of N-doped ZnO@g-C_3_N_4_ (1, 2, 4 and 6 wt%) and S-doped ZnO@g-C_3_N_4_ (1, 2, 4 and 6 wt%) nanocomposites were labelled as 1 NZCN, 2 NZCN, 4 NZCN, and 6 NZCN and 1 SZCN, 2 SZCN, 4 SZCN, and 6 SZCN, respectively. The dopant concentrations of N and S for the N-doped ZnO-g-C_3_N_4_ and S-doped ZnO-g-C_3_N_4_ nanocomposites were found to be 0.6 gm mL^−1^ and 0.06 gm mL^−1^, respectively.

### Characterization

Zeta potential analysis was done using a Zetasizer Nano ZS (Malvern Instruments). Typically, a fixed amount of each photocatalyst (20 mg) was ultrasonically dispersed in water for 30 min. The crystalline structures of the materials were identified using an X-ray diffractometer (ULTIMA IV, Rigaku) with Cu Kα radiation. The Fourier transform infrared spectra were studied on a PerkinElmer spectrometer (Spectrum-100) with the samples pressed in KBr. Thermogravimetric analysis (TG/DTA) was performed using a SDTQ600V209 Build 20 system maintaining a heating rate of 10 °C min^−1^. The specific surface area was calculated with an Autosorb-iQ (Quantachrome, USA) analyser by the BET method. UV-vis reflectance spectroscopy was performed on a Specord 210 Plus (Analytic Jena) instrument and BaSO_4_ was used as a reference. The morphologies and particle sizes of the as-prepared photocatalyst samples, along with the high-resolution images, were studied by FE-SEM on a Carl Zeiss SIGMA field emission microscope equipped with an energy dispersive X-ray spectroscope (Carl Zeiss SIGMA) and JM-2100 Plus (JEOL) transmission electron microscope. X-ray photoemission spectroscopy (XPS) data were obtained from an ESCALAB Xi^+^ (Thermo Fischer Scientific Pvt. Ltd. UK). Photoluminescence (PL) spectra were obtained using a fluorescence spectrophotometer (Horiba Scientific Fluorolog-3) at 325 nm.

### Photocatalytic measurement

The catalytic efficiencies of catalysts were examined by degrading crystal violet (CV) and brilliant green (BG) under visible light (light source: 90 W LED light with luminous intensity 8060 candela). In each test, a fixed amount (20 mg) of as-synthesized sample was mixed with CV (100 mL, 100 ppm) or BG (100 mL, 100 ppm) dye solution. The pH values of the suspension were adjusted by adding 0.1 M HCl or 0.2 M NaOH. The suspension was stirred in the dark for 30 minutes to confirm that the catalyst surfaces were saturated with dye solution before visible-light irradiation. A 3 mL sample was withdrawn from the reaction mixture every time and centrifuged (6000 rpm for 10 min) to separate essentially all the catalyst, followed by absorbance measurement. The concentrations of CV and BG were spectrophotometrically recorded by measuring the absorbances of CV and BG at 591.0 nm and 625.0 nm, respectively. The content ratios of Zn in the catalysts were analysed using inductively coupled plasma mass spectrometry (Agilent 7850 ICP-MS).

### Detection of reactive species

Various scavengers, EDTA, isopropyl alcohol, ascorbic acid and potassium iodide as scavengers for h^+^, ˙OH, ˙O_2_^−^ and both ˙OH and h^+^, respectively, were added into the CV and BG solutions before the addition of photocatalysts.^12^

## Conflicts of interest

There are no conflicts to delcare.

## Supplementary Material

RA-014-D3RA06488C-s001
