# Supplementary material for: Highly efficient visible-light induced N-doped ZnO@g-C3N4 and S-doped ZnO@g-C3N4 photocatalysts for environmental remediation
Source: RSC Adv. 2024 Jan 3;14(2):1156–68. doi: 10.1039/d3ra06488c (PMC10762517; doi:10.1039/d3ra06488c)
Supplement: RA-014-D3RA06488C-s001 [file RA-014-D3RA06488C-s001.pdf]

Electronic Supplementary Material (ESI) for RSC Advances.
This journal is © The Royal Society of Chemistry 2024

[illegible] nation

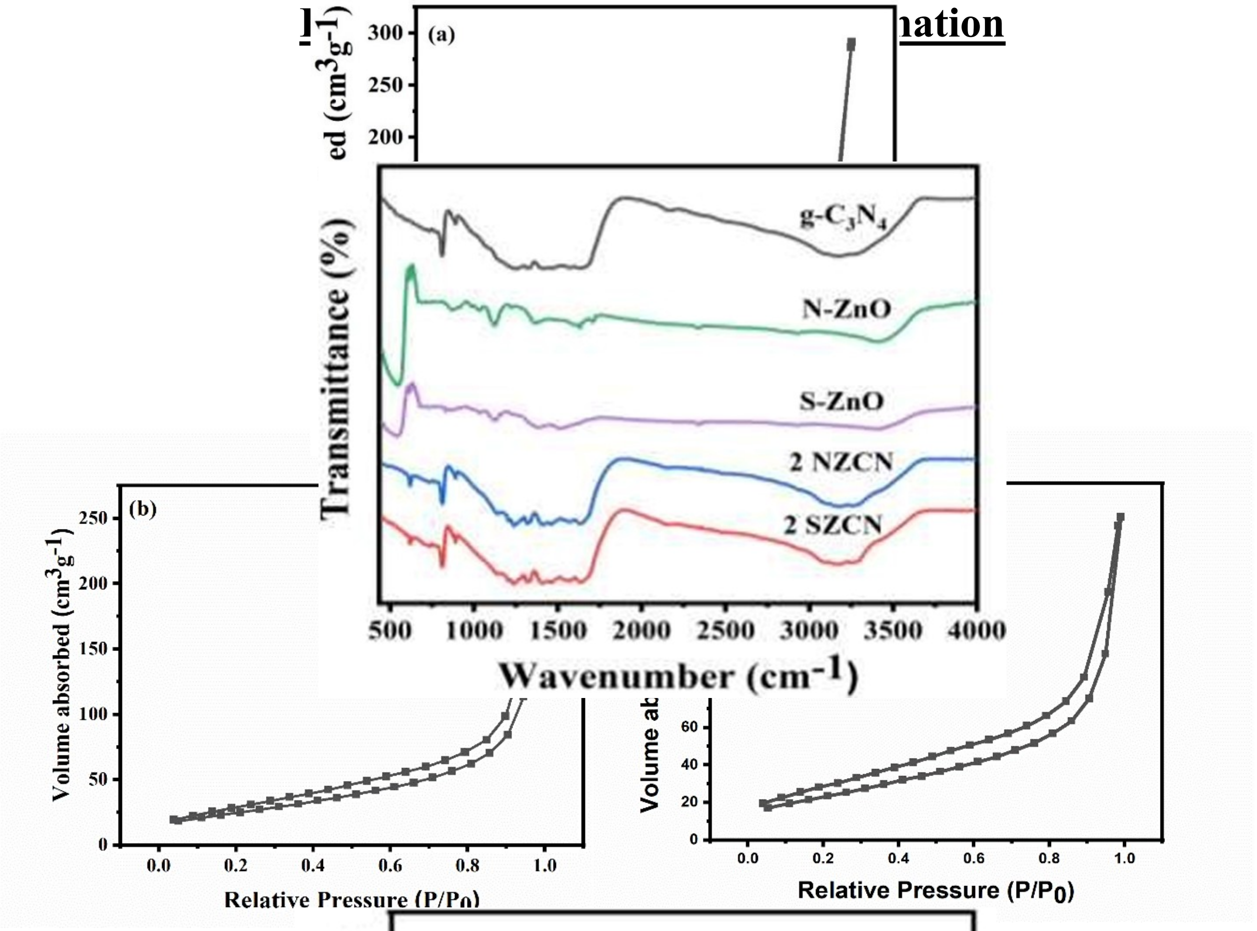

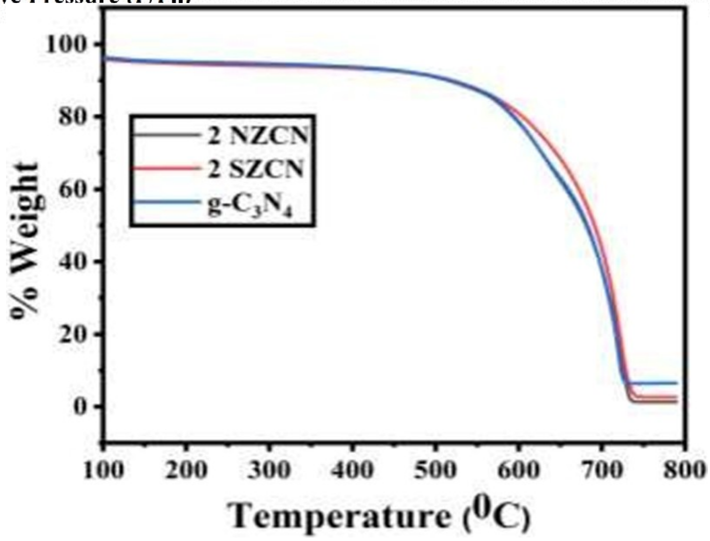

Figure S2. TG/DTA curves of g-C_3N_4, 2 NZCN and 2 SZCN nanocomposite

Figure S3. N_2 adsorption-desorption isotherm of (a) g-C_3N_4 (b) 2 NZCN (c) 2 SZCN nanocomposite

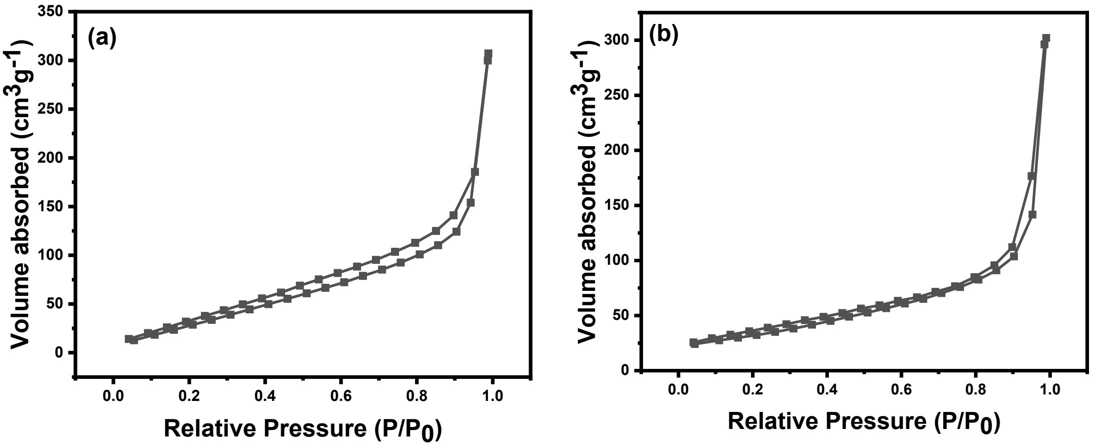

Figure S4. N_2 adsorption-desorption isotherm of (a) N-doped ZnO (b) S-doped ZnO.

Table S1 BET surface areas of as-prepared photocatalysts.

Photocatalyst	BET Surface Area (m^2g^{-1})
g-C_3N_4	66.5
N-ZnO	47.2
S-ZnO	33.3
1 NZCN	111.2
1 SZCN	108.0
2 NZCN	89.1
2 SZCN	84.2
4 NZCN	76.5
4 SZCN	68.0
6 NZCN	66.5
6 SZCN	63.1

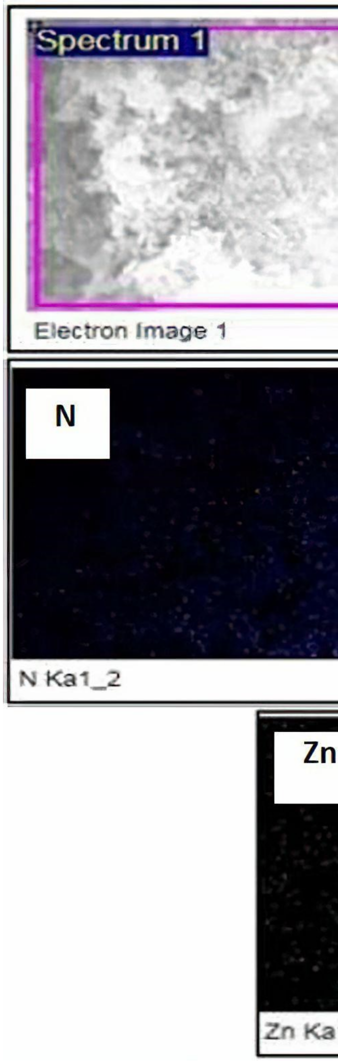

Figure S5. EDX elemental mapping of 2 NZCN nanocomposite.

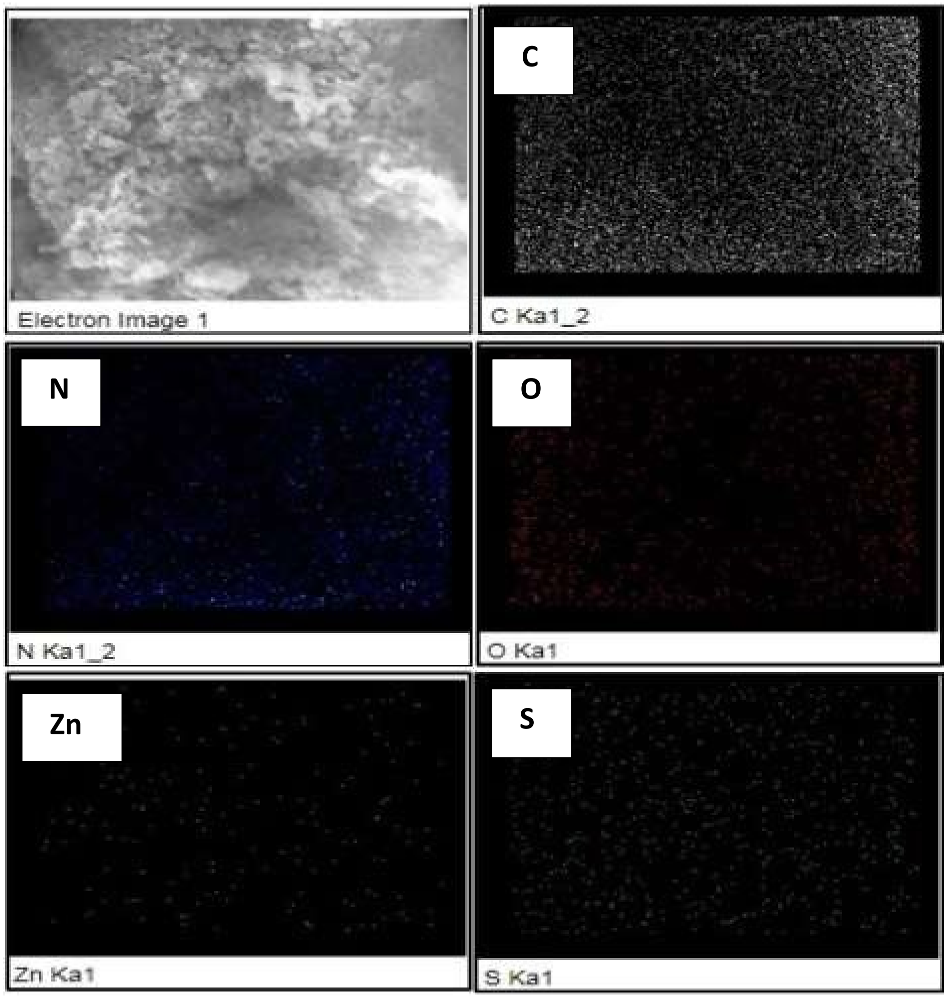

Figure S6. EDX elemental mapping of 2 SZCN nanocomposite.

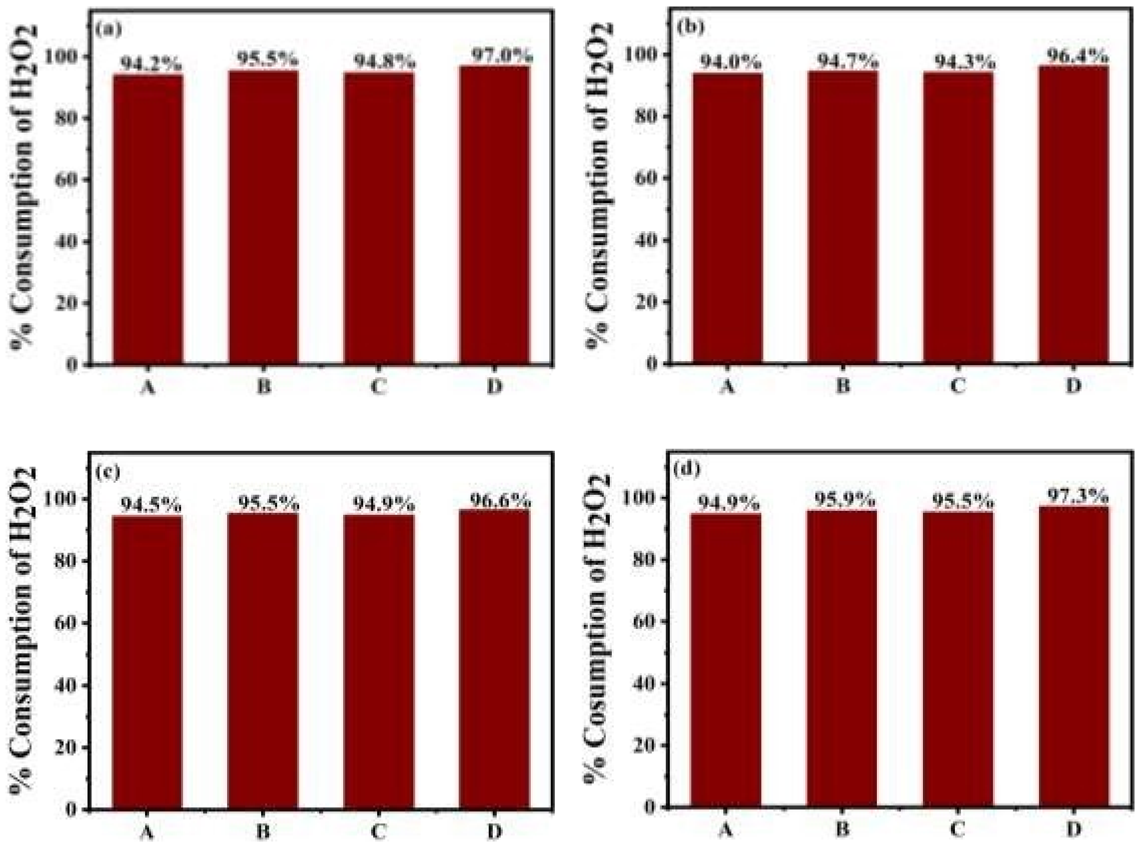

Figure S7. Consumption of H_2O_2 in photodegradation of (a) CV by 2 NZCN photocatalyst (b) CV by 2 SZCN photocatalyst (c) BG by 2 NZCN photocatalyst, and (d) BG by 2 SZCN photocatalyst under various conditions (A) H_2O_2, (B) H_2O_2/Vis, (C) Cat/H_2O_2, (D) Cat/H_2O_2/Vis.

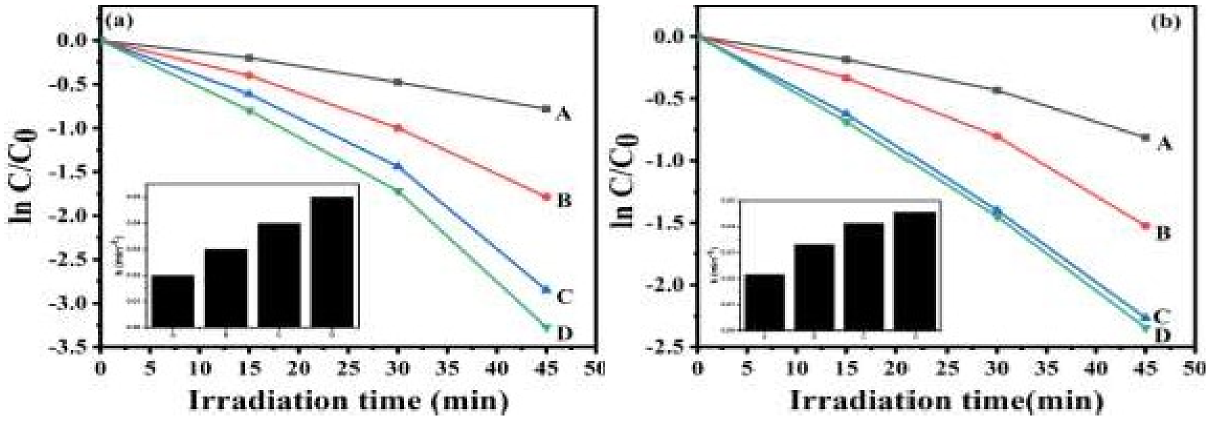
(a)
(b)
ln C/C0
Irradiation time (min)
Irradiation time(min)
A
B
C
D

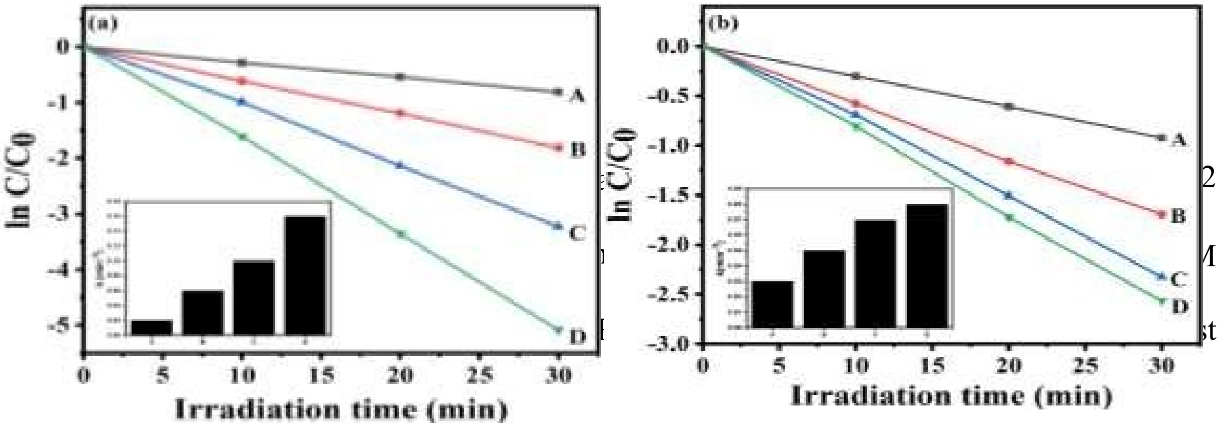
(a)
(b)
ln C/C0
Irradiation time (min)
A
B
C
D

Figure S9. Kinetics of photodegradation of (a) BG by 2 NZCN nanocomposite and (b) BG by 2 SZCN nanocomposite over different H_2O_2 concentration (A) 0.005 M (B) 0.01 M (C) 0.15 M (D) 0.10 M. Inset: degradation rate constant k. (Reaction conditions: [Dye] = 100 mg/ L, Catalyst = 200 mg/L, T = 25 ^{0}C, pH = 6).

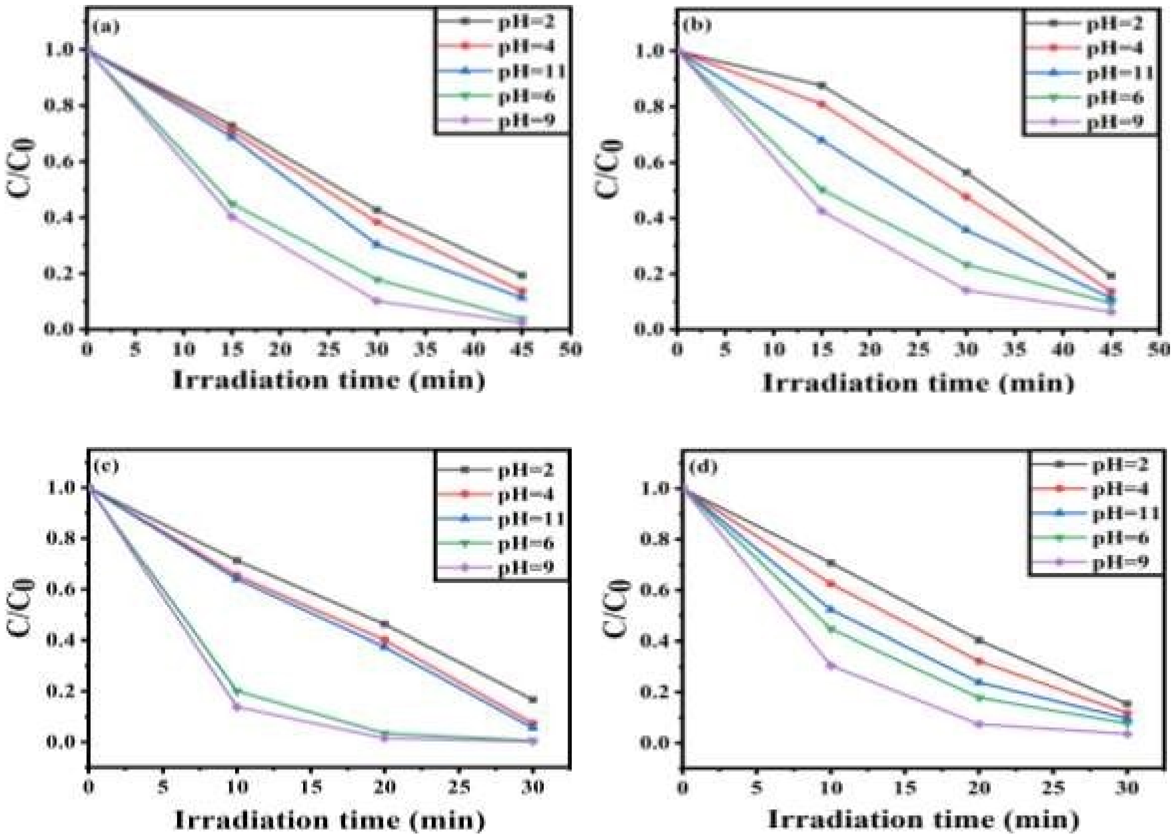

Figure S10. Influence of pH on photodegradation of (a) CV by 2 NZCN nanocomposite (b) CV by 2 SZCN nanocomposite (c) BG by 2 NZCN nanocomposite and (d) BG by 2SZCN nanocomposite (Reaction conditions: [Dye] = 100 mg/ L, Catalyst = 200mg/L,T = 25 ^{0}C).

Characterization of recovered catalyst:

The 2 NZC[illegible]ized by FE-SEM ar[illegible]ows no obvious dif[illegible]y of the photocataly[illegible]

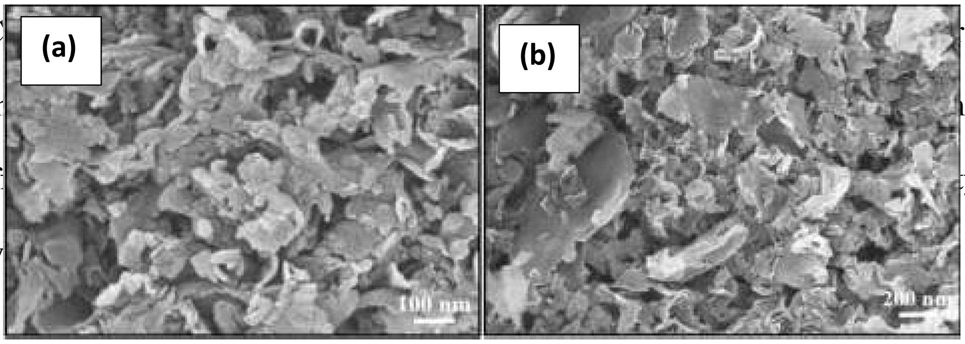

Figure S11. FE-SEM image of recovered (a) 2 NZCN nanocomposites (b) 2 SZCN nanocomposite.

In order to investigate the chemical composition variation of the 2 NZCN and 2SZCN nanocomposite after the photodegradation reaction, XPS measurements were done on (ESCALAB Xi+, Thermo Fisher Scientific Pvt. Ltd., UK) which shows no noticeable change even after seventh catalytic cycle, indicating their high chemical stability in photodegradation process as depicted in Fig. S11 (a, b, c, d, e) and Fig. S12 (a, b, c, d, e, f) respectively. The XPS survey spectrum of 2NZCN nanocomposite shows the presence of C 1s, N 1s, O 1s and N 2p elements as depicted in Fig. S11 (a). Similarly, the XPS survey spectrum of 2SZCN nanocomposite indicates the presenceof C 1s, N 1s, O 1s, N 2p and S 2p elements as depicted

in Fig. S12 (a).

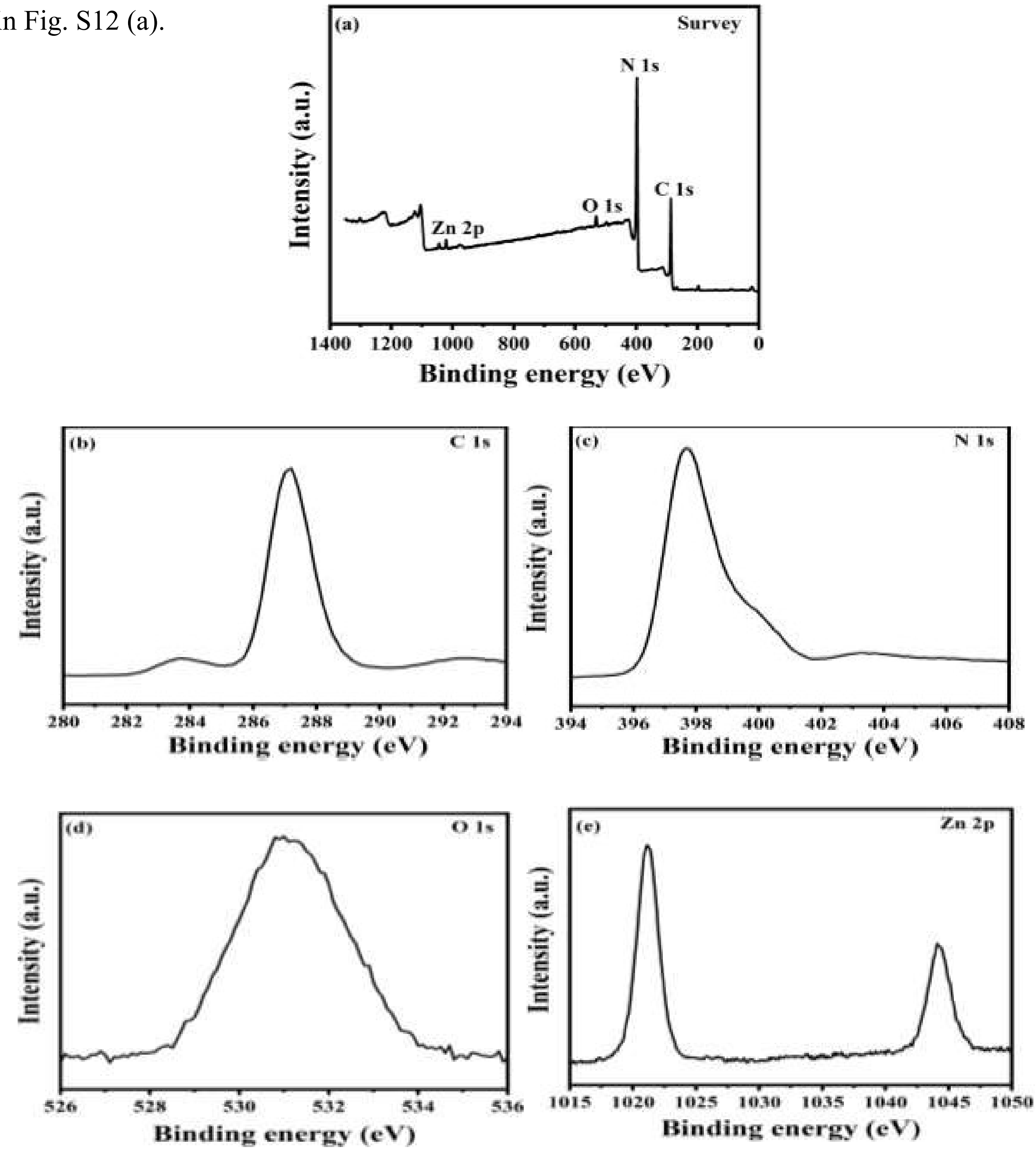

Figure S12. (a) XPS survey spectra of recovered 2 NZCN nanocomposite, (b) C 1s XPSspectra, (c) N 1s XPS spectra, (d) O1s XPS spectra and (e) Zn 2p XPS spectra of recovered 2 NZCN

nanocomposite.

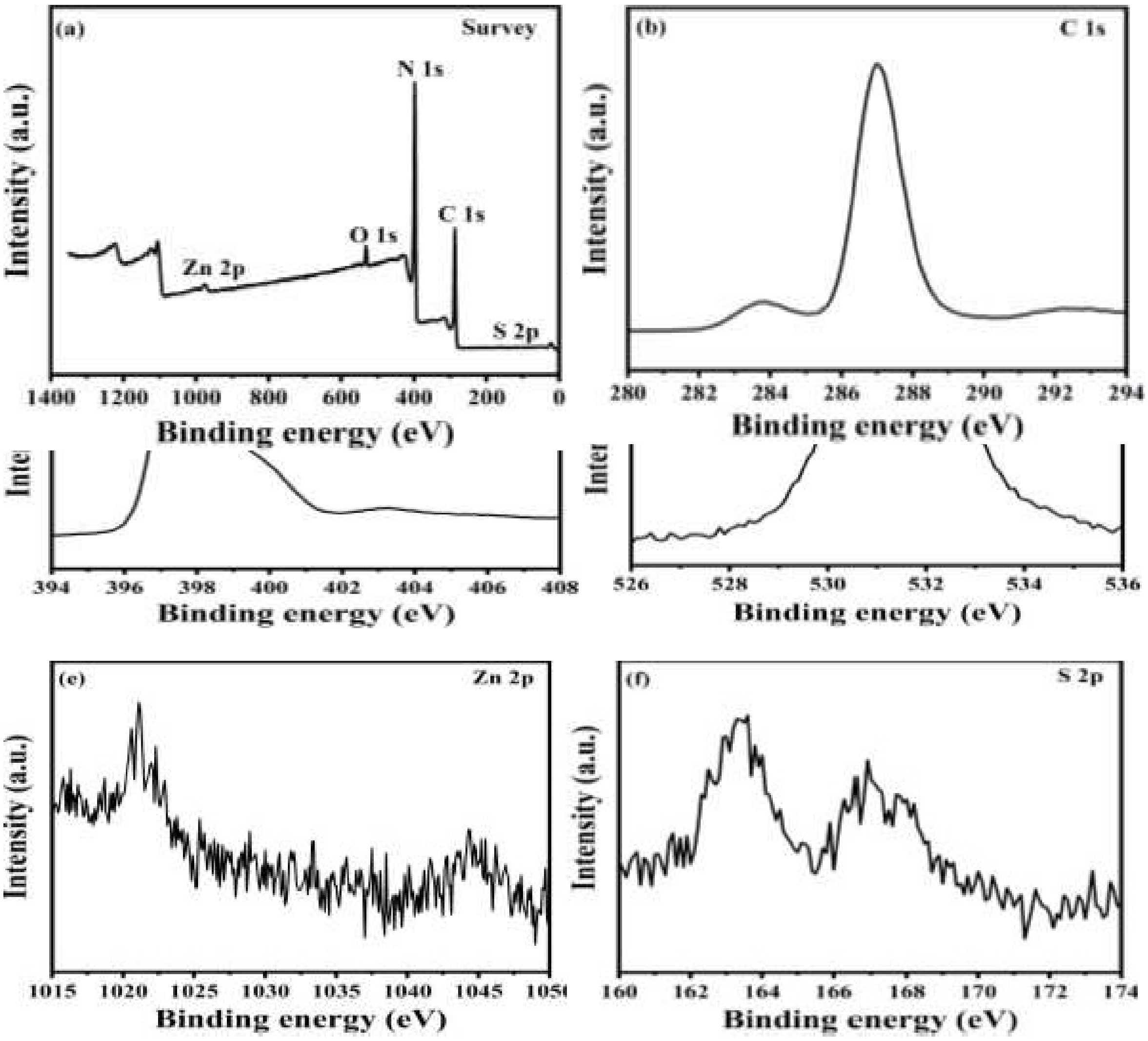

Figure S13. (a) XPS survey spectra of recovered 2 SZCN nanocomposite, (b) C 1s XPS spectra, (c) N 1s XPS spectra, (d) O1s XPS spectra, (e) Zn 2p XPS spectra and (f) S 2p XPS spectra of

recovered 2 NZCN nanocomposite.

Table S2. Comparison of different photocatalysts towards photocatalytic degradationCrystal violet and Brilliant green.

**Photocatalytic degradation of Brilliant green dye molecule**					
**Photocatalyst**	**Conc.(ppm)**	**Time (min)**	**% Degradation**	**Light source**	**Reference**
N-ZnO-GO	20	90	100	Visible	[1]
Ag_8SnS_6	10	90	92.3	Tungsten	[2]
Cu- and Ag-doped ZnO	10	180	100	UV	[3]
$(ZnO\text{-}TiO_2)$ -β-CD	20	180	99	Visible	[4]
Mn-CDSe QDS	10	90	86.5	Sunlight	[5]
$Sr\text{-}TiO_2$	25	60	96	UV	[6]
$Fe_3O_4/CdS\text{–}ZnS$	10	60		Visible	[7]
CeO_2/Zeolite-NaX	10	180	94.8	Visible	[8]
ZnO	20.7 µM	60	94.3	MW/UV	[9]
ZnS quantum dots	10	80	88	Solar	[10]
N-doped $ZnO@g\text{-}C_3N_4$<br>S-doped $ZnO@g\text{-}C_3N_4$	**100**<br>**100**	**30**<br>**30**	**99.3**<br>**92.3**	**Visible**	**This work**
**Photocatalytic degradation of Crystal violet dye molecule**					
**Photocatalyst**	**Conc.(ppm)**	**Time (min)**	**% Degradation**	**Light source**	**Reference**
Bi_2WO_6	50	4320	99.8	UV	[11]
BiO_xCl_y/BiO_mI_n	10	720	99.5	Visible	[12]
Cu doped ZnO	10	210	100	UV-Visible	[13]
Ag^+ doped TiO_2	20	600	88	Solar	[14]
ZnO	10	80	96	UV	[15]
N-doped $ZnO@g\text{-}C_3N_4$<br>S-doped $ZnO@g\text{-}C_3N_4$	**100**<br>**100**	**45**<br>**45**	**96.2**<br>**90.4**	**Visible**	**This work**

References

1. C.N. Peter, W.W Anku, R. Sharma, G.M. Joshi, S.K. Shukla and P.P. Govender, Ionics., 2019, 25, 327-339.

2. B.H. Shambharkar and A.P. Chowdhury, RSC Adv, 2016, 6, 10513–10519.

3. A. Gnanaprakasam, V. Sivakumar and M. Thirumarimurugan, Water Sci Technol, 2016, 74, 1426–1435.

4. P. Velusamy and G. Lakshmi, India J Chem, 2017, 56, 43–49.

5. N. Thirugnanam and D. Govindarajan, Int J Recent Sci Res, 2016, 7, 13377–13382.

6. S. Sood, A. Umar, M.S. Kumar, A.S.K. Sinha and S.K. Kumar, Ceram Int., 2015, 41, 3533–3540.

7. F. Soltani-nezhad, A. Saljooqi, A. Mostafavi and T. Shamspur, Ecotoxicol. Environ. Safe., 2020, 189, 109886.

8. 37. P. Huo, M. Zhou, Y. Tang, X. Liu, C. Ma, L. Yu and Y. Yan, J. Alloys Compd. 2016, 670, 198-209.

9. V.L. Gole and A. Priya, J. Water Process Eng.., 2017, 19, 101-105.

10. S. Kaur, S. Sharma, A. Umar, S. Singh, S.K. Mehta and S.K. Kansal, Superlattices Microstruct., 2017, 103, 365-375.

11. S. Kumar, V. Sharma, K. Bhattacharyya, V. Krishnan, Mater. Chem. Front., 2017, 1, 1093-1106.

12. Y-R. Jianga, H-P. Lina, W-H Chungb, Y-M. Daia, W-Y. Linb and C-C. Chena, J. Hazard. Mater., 2015, 283, 787–805.

13. M. Mittal, M. Sharma and O.P. Pandey, Sol. Energy, 2014, 110, 386–397.

14. C. Sahoo, A.K. Gupta and A. Pal, Dyes Pigm., 2005, 66, 189-196.

15. S. Ameen, M. S. Akhtar, M. Nazim, H-S. Shin, Mater. Lett., 2013, 96, 228–232.
